# Estimating Protein Conformational States from High-Speed AFM Images with Molecular Dynamics and Deep Learning

**DOI:** 10.1021/acs.jcim.6c00142

**Published:** 2026-04-04

**Authors:** Katsuki Sato, Yui Kanaoka, Tomoya Tsukazaki, Takayuki Uchihashi, Takaharu Mori

**Affiliations:** † Department of Chemistry, Faculty of Science, 26413 Tokyo University of Science, Shinjuku-ku, Tokyo 162-8601, Japan; ‡ Department of Physics, Graduate School of Science, 12965 Nagoya University, Furo-cho, Chikusa-ku, Nagoya, Aichi 464-8602, Japan; § 12708 Nara Institute of Science and Technology, Ikoma, Nara 630-0192, Japan; ∥ Exploratory Research Center on Life and Living Systems (ExCELLS), National Institutes of Natural Sciences, 5-1 Higashiyama, Myodaiji, Okazaki, Aichi 444-8787, Japan; ⊥ Institute for Glyco-Core Research (IGCORE), Nagoya University, Furo-Cho, Chikusa-Ku, Nagoya, Aichi 464-8601, Japan; # Quantum-Based Frontier Research Hub for Industry Development (Q-BReD), Nagoya University, Nagoya 464-8601, Japan

## Abstract

High-speed atomic force microscopy (HS-AFM) is a powerful technique for visualizing protein dynamics in real time at the single-molecule level and has enabled direct observation of diverse biomolecular processes such as protein conformational changes, enzymatic reactions, and protein–protein interactions. Despite these advantages, HS-AFM imaging often suffers from substantial noise and limited spatial resolution, which complicates the reliable identification of detailed protein conformational states. To address these limitations, we introduce DeepAFM, a framework that integrates deep learning with molecular dynamics (MD) simulations to estimate protein conformational states while denoising AFM images. The model is trained on simulated AFM images generated from MD snapshots, incorporating realistic noise to mimic experimental conditions, including temporal lag effects between line scans. As a case study, we apply DeepAFM to the membrane protein SecYAEG–nanodisc complex, in which SecA undergoes conformational transitions between closed and wide-open states. The trained model preferentially attends to regions in the input images corresponding to large-scale domain motions of SecA, thereby increasing robustness to noise-induced overfitting compared with conventional rigid-body and flexible fitting. By effectively denoising experimental HS-AFM images, DeepAFM estimates the dominant conformational states of the protein, in agreement with independent experimental observations. DeepAFM provides a deep-learning-assisted analysis strategy for the interpretation of noisy HS-AFM data.

## Graphical abstract

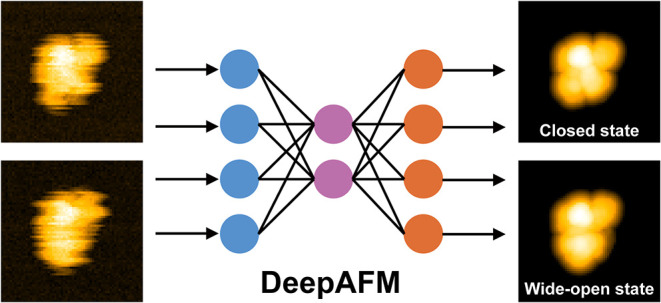

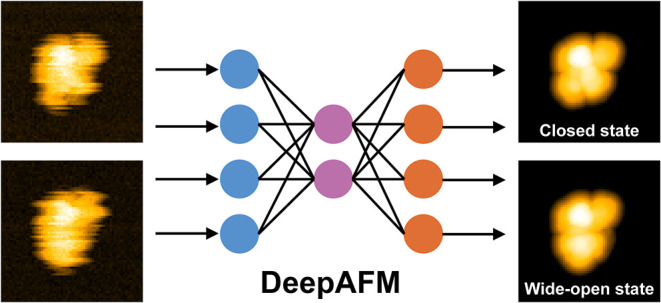

## Introduction

High-speed atomic force microscopy (HS-AFM) is a powerful tool for real-time observation of protein dynamics at the single-molecule level. By rapidly scanning the surface of target proteins with the cantilever and measuring height variations, HS-AFM generates dynamic movies of protein motions with millisecond resolution, providing valuable insights into protein conformational changes, enzymatic reactions, and molecular interactions.^1^ This technique has enabled the direct observation of a range of biological phenomena, such as the walking motion of myosin on actin filaments,^2^ DNA cleavage mediated by Cas9,^3^ large-scale conformational changes in the AMPA receptor,^4^ and substrate transport through the SecY channel.^5^ Despite its high vertical resolution (∼0.1 nm), the lateral resolution of HS-AFM remains limited (2–3 nm).^6^ Additionally, the temporal lag during line scanning can cause distortion of molecular structures observed in AFM images,^6^ making it challenging to accurately interpret protein conformational states.

Analysis of protein conformational states in HS-AFM images has conventionally relied on rigid-body and flexible fitting approaches. In rigid-body fitting, a three-dimensional (3D) protein structure is aligned to an AFM image by rigid-body translation and rotation to maximize similarity between simulated and experimental images.^7–9^ Flexible fitting, in contrast, allows conformational changes and is typically conducted with molecular dynamics (MD) simulations or a normal-mode analysis (NMA). MD-based methods use a molecular mechanics force field with a biasing potential incorporating a similarity metric, such as the correlation coefficient (c.c.),^10^ whereas NMA-based methods move atoms along low-frequency normal mode vectors to enable large-scale structural changes for fitting.^11–13^ Recently, a data assimilation framework has also been proposed for sequential inference of protein dynamics by integrating HS-AFM data with coarse-grained MD simulations while accounting for the temporal lag in AFM measurements.^14–16^ In general, fitting 3D structures to noisy 2D AFM images can lead to overfitting, depending on the algorithm’s robustness. Therefore, it is important to carefully evaluate whether the resulting models reflect genuine structural features rather than artifacts from the experimental noise.

In recent years, deep learning (DL) has emerged as a powerful tool for image analysis, which tackles complex tasks such as classification, noise reduction, and super-resolution. For classification, high-performance DL models including ResNet,^17^ AlexNet,^18^ and Vision Transformer (ViT)^19^ have been developed. For noise reduction and super-resolution, DL models based on autoencoders, such as U-Net^20^ and DnCNN,^21^ are commonly used. These techniques find applications in biology and chemistry, for example, in cryo-electron microscopy (cryo-EM),^22^ fluorescence imaging,^23^ and AFM.^24,25^ Specifically for AFM image analysis, DL has been applied to diverse problems, such as classifying small organic molecules,^26^ achieving super-resolution imaging of DNA,^27^ denoising amyloid fibril images,^28^ analyzing the dynamics of protein self-assembly,^29^ and determining structures of RNA conformers.^30^ Prediction of AFM tip shapes has also been examined.^31^ These advances highlight the potential of DL as a powerful approach for interpreting protein dynamics in noisy HS-AFM images.

In this study, we introduce DeepAFM, an algorithm that combines DL with MD simulations to estimate protein conformational states while simultaneously denoising the images, specifically by reducing image distortions caused by temporal scanning lag. We apply DeepAFM to HS-AFM images of the membrane protein SecYAEG–nanodisc complex,^32^ which undergoes conformational transitions between closed and wide-open conformations. The algorithm not only effectively denoises the images but also estimates protein conformational states consistent with observations from previous independent experiments.^5^ We further discuss the potential of transfer learning as a strategy to extend DeepAFM to other systems. We expect that DeepAFM provides an effective alternative to conventional methods for analyzing protein dynamics in HS-AFM data.

## Results

### Algorithm of DeepAFM

An overview of DeepAFM is shown in Figure 1A. Conformational changes of the target protein are first sampled by MD simulations, and simulated AFM images are generated from each snapshot in both “noise-free” and “noise-added” forms to build a data set for DL. The noise-free images are hereafter referred to as ground-truth images. Principal component analysis (PCA) and clustering are then applied to the MD trajectory data to assign a conformational state (cluster label) to each snapshot. These images, paired with their corresponding ground-truth labels (hereafter referred to as ground-truth states), are used to train a neural network that simultaneously performs image denoising and protein state classification. For the neural network, we used a ViT-based multitask deep autoencoder (AE) (see Materials and Methods and Figure S1 and Table S1 for details).^19^ Finally, the trained model is applied to experimental HS-AFM images to both denoise them and identify protein conformational states.

**Figure 1:**
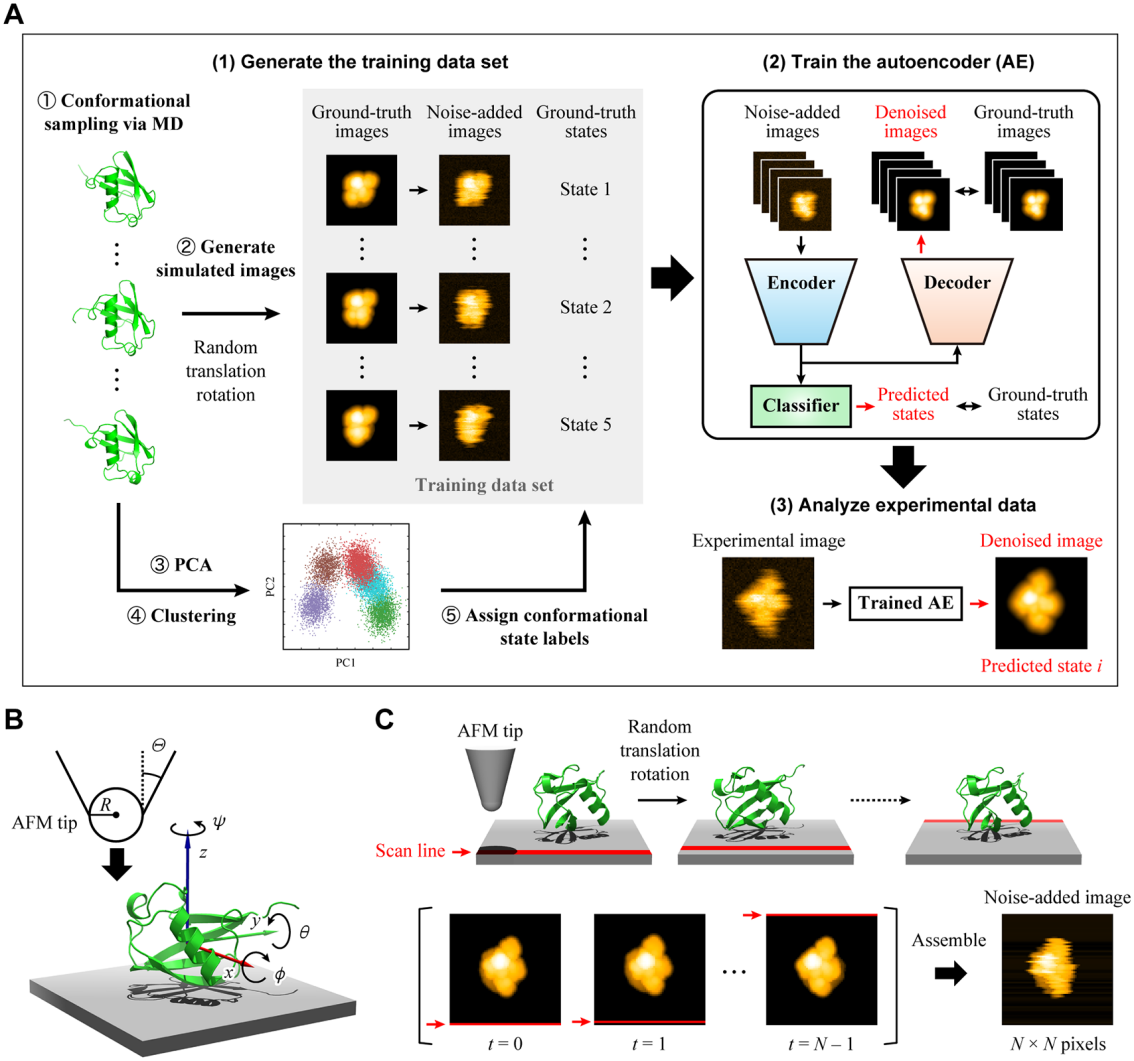
Overview of the DeepAFM algorithm. (A) Workflow for generating the training data set, training the autoencoder (AE), and applying the trained AE to experimental data. (B) Definition of parameters randomly varied during simulated image generation, including AFM tip probe radius *R*, cone half-apex angle Θ, and protein orientation (ϕ, θ, ψ). The stage height was defined as the minimum *z*-coordinate of the protein. (C) Procedure for generating noise-added images by emulating distortions caused by temporal scanning lag in HS-AFM measurements. After completing a scan along the *x*-direction (red), the probe shifts in the *y*-direction to the next line. At each line scan, the protein’s lateral position and orientation are randomly displaced from their initial values (*x*_0_, *y*_0_, ϕ_0_, θ_0_, ψ_0_) at *t* = 0 by Δ*x*, Δ*y*, Δϕ, Δθ, and Δψ.

AFM imaging is influenced by various factors, including the size and geometry of the AFM tip, the orientation and lateral position of the protein relative to the stage, and the measurement noise. In typical protocols for generating simulated AFM images, collision detection–based approaches have been widely employed (Figure 1B).^10,33,34^ For each pixel, the tip is moved vertically toward the target molecule and the height is defined as the lowest tip position at which steric contact occurs with the molecular surface. The tip is modeled as a cone capped by a sphere with probe radius *R* and cone half-apex angle Θ. The protein orientation is parametrized by rotations about the *x*-, *y*-, and *z*-axes (ϕ, θ, and ψ, respectively), and the lateral position is parametrized by translations along the *x*- and *y*-axes (*x* and *y*, respectively). To generate a training data set, these parameters were randomly varied within empirically determined ranges to reproduce the appearance of experimental images. In this study, we first generated simulated images without any added noise (ground-truth images).

To generate noise-added images, we not only applied additive white noise but also explicitly accounted for the asynchronous nature of HS-AFM scanning (Figure 1C). Conventional protocols for generating simulated images typically use a single, instantaneous structural snapshot of the target protein, assuming a static structure throughout the scan. In actual experiments, however, the scanning process is inherently asynchronous: each pixel is measured sequentially with a time interval on the order of microseconds. Within this brief yet significant time window, proteins can undergo structural changes, translational movements, and rotational fluctuations.^6^ To better emulate these conditions, we approximately incorporated Brownian motion into the image generation process (Figure 1C, upper panels). Specifically, for each scanned line, we randomly updated the lateral position and orientation of the target protein. These sampled lines were then assembled to produce a single image (Figure 1C, lower panels). While the internal conformation of the protein was maintained throughout the scanning process, the approach successfully produced noisy images closely resembling real HS-AFM images (Figure S2).

### Target System and Preparation of the Training and Test Data Sets

DeepAFM is designed as a general framework applicable to a wide range of systems. In this study, prior to extending its application to diverse targets, we aimed to evaluate its intrinsic performance. To this end, we selected the *Thermus thermophilus* SecYAEG complex embedded in a nanodisc (ND) (Figure 2A, left panels) and analyzed its HS-AFM images. SecYAEG is a membrane protein complex consisting of the protein-conducting channel SecYEG and the motor protein SecA ATPase.^35–37^ SecA consists of several domains, including PPXD, NBD1, NBD2, and HWD, and undergoes conformational transitions between the closed, open, and wide-open states, primarily through rigid-body movements of the PPXD domain (Figure 2A, right panels).^38,39^ Haruyama et al. employed HS-AFM to visualize the *T. thermophilus* SecYAEG–ND complex. They observed two distinct orientations of the complex: an *end-up* orientation, in which the ND plane is parallel to the AFM stage, and a *side-on* orientation, in which it is perpendicular.^32^ Recently, Kanaoka et al. successfully captured protein translocation events using HS-AFM in the side-on orientation.^5^ They found that, in the absence of a translocating preprotein, SecA predominantly adopts the wide-open state, whereas, in the presence of a preprotein, it undergoes conformational transitions between the closed and wide-open states, as inferred from height changes in the HS-AFM images. However, detailed characterization of SecA conformations remains challenging due to the limited spatial resolution and intrinsic noise of AFM imaging; in particular, interpretations based on end-up HS-AFM images are still limited. In this study, we focus on the end-up HS-AFM images observed in the absence of a preprotein.

**Figure 2:**
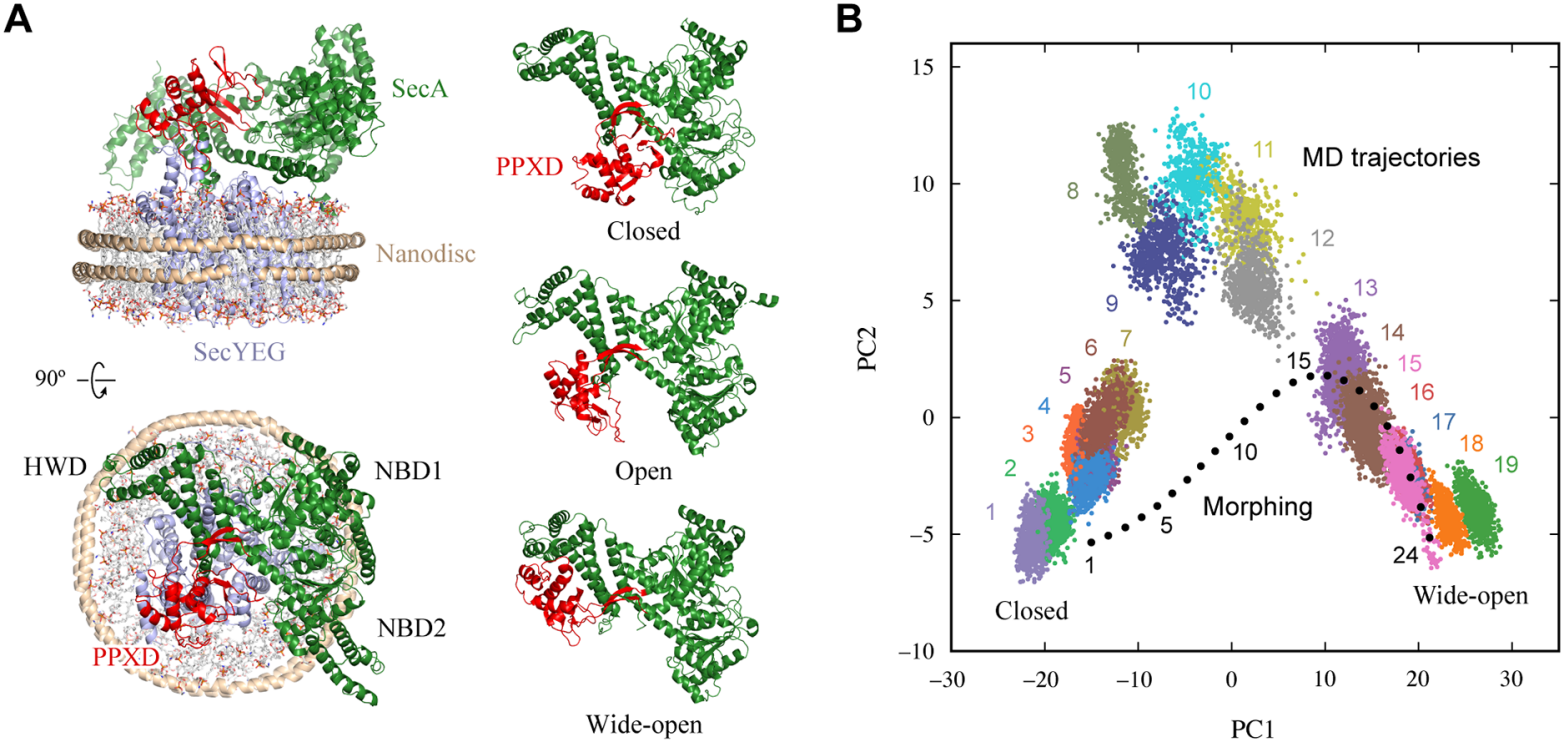
Structural overview and conformational changes of the SecYAEG–ND complex. (A) Left panels: SecYAEG–ND complex in the closed state of *Thermus thermophilus*, presented in side view (top) and top view (bottom). SecYEG is colored light blue, and SecA is colored green, with the PPXD domain highlighted in red. Right panels: SecA in *Bacillus subtilis*: closed (top, PDB: 6ITC), open (middle, PDB: 1TF5), and wide-open (bottom, PDB: 1M6N) states. (B) PCA of the MD trajectories, in which conformational states were clustered into 19 groups. The morphing trajectory, consisting of 24 snapshots, is projected onto the same space and shown in black.

There are various MD-based methods for the conformational sampling of proteins, and the optimal approach depends on the system under investigation. In this study, we first performed a targeted MD (TMD) simulation^40^ of the SecYAEG–ND complex to induce the conformational transition of SecA from the closed to the wide-open state. Subsequently, we performed restart MD simulations from selected intermediate structures to explore a broader conformational space (see Materials and Methods for details). We then applied PCA and clustering to the MD trajectories (14,000 snapshots in total) and identified 19 representative conformational states (colored scatter plots in Figure 2B). Within these clusters, the mean RMSD of the structures was 1.7 Å (Figure S3). Cluster labels were assigned from 1 to 19 such that they increase with the conformational transition from the closed to the wide-open state, based on the average PC1 values of the clusters. As a result, for pairs of clusters separated by one state, the mean intercluster RMSD was 2.9 Å. Note that the optimal number of clusters generally depends on the conformational complexity of the target system. Since RMSD values greater than ∼3 Å typically reflect substantial global conformational changes, the use of 19 clusters appears sufficient to capture the meaningful conformational variations present in this data set. From these MD trajectories, we generated 6.4 million images for training and 0.8 million images for validation, for both ground-truth and noise-added images.

To generate these simulated images, we randomly varied the AFM probe radius (*R*), cone half-apex angle (Θ), protein lateral position and orientation parameters (*x*, *y*, ϕ, θ, ψ), and the standard deviation of white noise (σ_*N*_) (see Materials and Methods). To approximately introduce Brownian motion in noise-added image generation, the protein parameters were displaced from their randomly generated initial values (*x*_0_, *y*_0_, ϕ_0_, θ_0_, and ψ_0_) by Δ*x*, Δ*y*, Δϕ, Δθ, and Δψ. These displacements were sampled from zero-mean normal distributions, Δ*p*_*i*_ ∼ *N*(0, *s*σ_*i*_^2^), where *p*_*i*_ ∈ {*x*, *y*, ϕ, θ, ψ} and *s* is a scaling factor that scales the variance σ_*i*_^2^. To avoid unrealistically large perturbations, the displacements were further restricted such that Δ*r* ≤ *w*Δ*r*_max_, |Δϕ| ≤ *w*Δϕ_max_, |Δθ| ≤ *w*Δθ_max_, and |Δψ| ≤ *w*Δψ_max_, where Δ*r* = √(Δ*x* ^2^ + Δ*y* ^2^) and *w* is a truncation scaling factor. In this study, to generate a reference training data set, we used *s* = 1, *w* = 1, σ_*x*_^2^ = σ_*y*_^2^ = 0.81 nm^2^, σ_ϕ_^2^ = σ_θ_^2^ = 6.25°^2^, σ_ψ_^2^ = 25°^2^, Δ*r*_max_ = 1.2 nm, Δϕ_max_ = Δθ_max_ = 5°, and Δψ_max_ = 10°. These ranges were empirically chosen to ensure that noise-added images resembled experimental ones. In addition, zero-mean-white noise with a standard deviation σ_*N*_ randomly sampled from 0.0 to 0.3 nm in 0.01 nm increments was added to the images. All simulated images were generated at a resolution of 36 × 36 pixels with a pixel size of 0.8 nm to match the experimental AFM images of the SecYAEG–ND complex.

To obtain additional structural snapshots, we performed morphing calculations.^41^ Morphing is a procedure that generates a structural transition pathway by interpolating between an initial and a final structure.^42^ The algorithm used in this study, Morphit Pro,^41^ interpolates interatomic distances and reconstructs atomic coordinates using multidimensional scaling. Using this approach, SecA in the SecYAEG–ND complex was transformed from the closed state to the wide-open state. The resulting trajectory consisted of 24 snapshots. As shown by the black plots in Figure 2B, which project the morphing trajectory onto the PCA space, Snapshots 1–5 moved away from the MD-sampled conformational region, Snapshots 6–15 approached another MD-sampled region, and Snapshots 16–24 overlapped with the MD ensemble. Note that in this morphing calculation, the end point structures are preserved exactly. The deviation of Snapshot 1 from State 1 arises because MD trajectories from the equilibration stage were excluded when constructing the MD ensemble (see Materials and Methods). The RMSD between Snapshot 1 and the cluster center of State 1 was 3.9 Å. These snapshots were used to generate a separate test data set of simulated images, which were employed to evaluate the robustness of DeepAFM under conditions in which MD simulations failed to fully capture the conformational diversity present in AFM images.

### Performance of DeepAFM for Simulated AFM Images

We first assessed the performance of the trained AE using simulated test images that contained no white noise. These comprised 0.8 million images generated by randomly selecting MD snapshots and assigning protein orientations under the same conditions as those used for the training data. Figure 3A­(a) presents three representative successful examples (tests 1–3), showing the input, denoised, and ground-truth images from left to right for each case. The denoised images exhibited effective noise reduction, while preserving the overall shape and size of the target protein. The locations of high-intensity regions were well preserved and closely matched those in the ground-truth images. The height profiles of the denoised images were in close agreement with the ground-truth profiles (Figure S4A). The mean-absolute errors (MAEs) between the denoised and ground-truth images were 0.127, 0.101, and 0.094 nm for tests 1–3, respectively. Figure 3A­(b) presents attention rollout maps derived from the classifier’s self-attention layers and overlaid on the input images. These maps visualize the spatial regions to which the AE model attended most strongly during classification. Across tests 1–3, the trained AE model predominantly focused on the PPXD domain of SecA or its surrounding regions. This observation suggests that the AE model can highlight regions associated with domain motions within the input image, thereby providing useful information about the relative positions and orientations of the protein domains.

**Figure 3:**
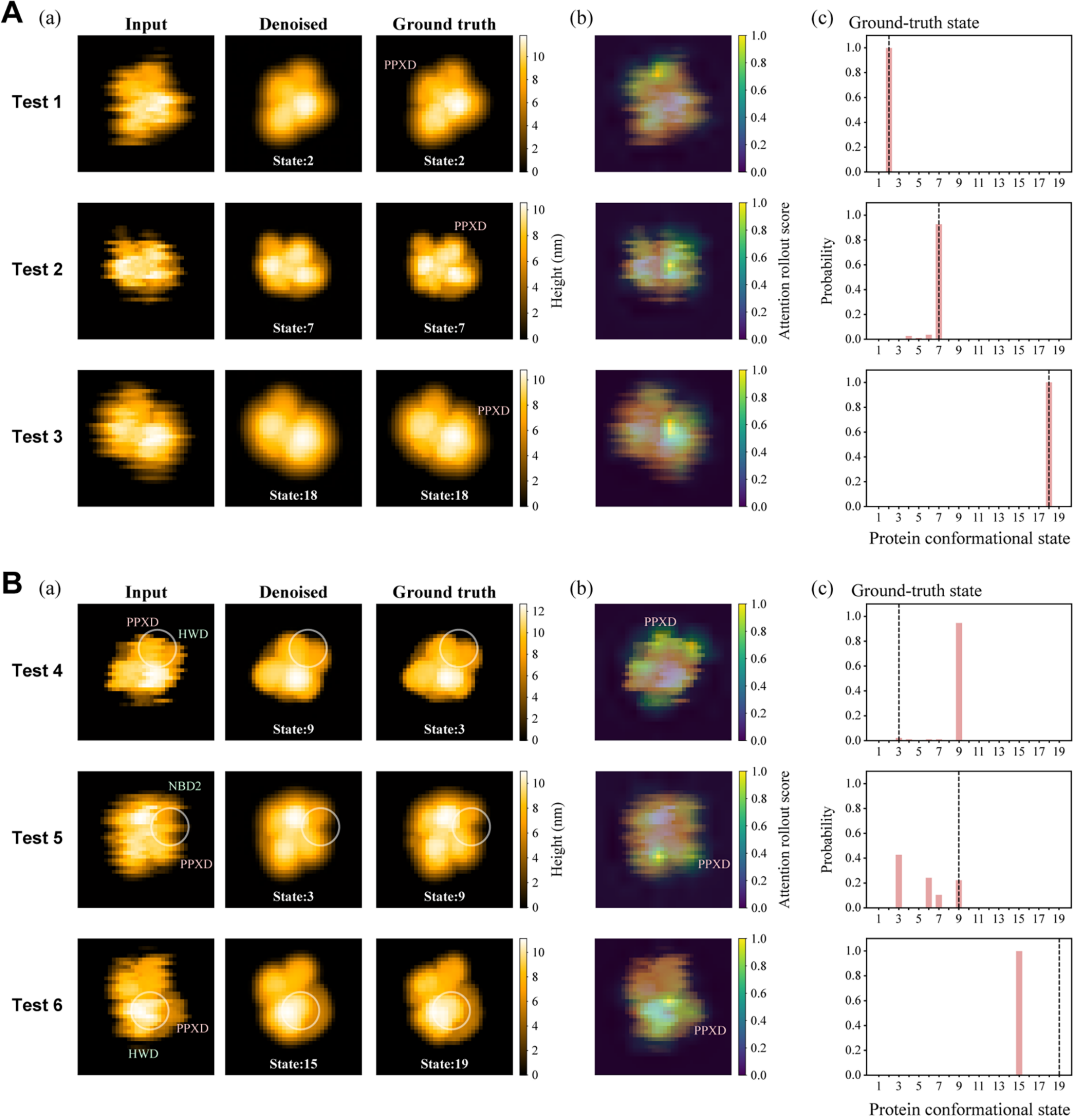
Performance of the trained AE on the test data set. (A) Successful examples and (B) unsuccessful examples. (a) From left to right: input, denoised, and ground-truth images. (b) Visualization of the attention rollout score overlaid on the input images. (c) Probability distributions of the classified conformational states, where the ground-truth state is indicated by a vertical dashed line.

The estimated conformational states were also consistent with the ground-truth states. Here, each estimated state corresponds to the conformational state with the highest probability among the 19 predefined states. As shown in Figure 3A­(c), the output probabilities exhibited a single peak at the ground-truth state, indicating high confidence in the correct classification. Across the 0.8 million test images, the exact-match accuracy was 93.4%, which increased to 98.1% when a tolerance of the ±1 state was allowed (Figure S5A). For these 0.8 million test images, the average MAE between the predicted and ground-truth images was 0.128 nm. Once trained, our AE model achieves both effective denoising of AFM images and high accuracy in classifying protein conformational states.

Despite the overall high accuracy, a small subset of classifications showed substantial errors. Among the 0.8 million test images, 699 cases deviated from the correct state by four or more states (corresponding to an effective RMSD of 4.4 Å or higher) (Figure S5A). Representative examples of such misclassifications are shown in Figure 3B (tests 4–6). In these cases, the denoised images resembled the ground-truth images [Figure 3B­(a)], and their MAEs were small (0.122, 0.109, and 0.125 nm for tests 4, 5, and 6, respectively). In addition, the trained AE model predominantly attended to the PPXD domain of SecA [Figure 3B­(b)]. However, the output probability distributions were either broad (test 5) or single-peaked but substantially shifted from the ground-truth state (tests 4 and 6) [Figure 3B­(c)]. These misclassifications were likely caused by line-scan noise affecting features that are important for recognizing the conformational states of SecA, including the groove between the PPXD and NBD2 or HWD domains (indicated by circles in Figure 3B­(a); see also height profiles in Figure S4B). As a result, the AE model may have failed to capture critical image features, resulting in reduced denoising performance and instability in conformational state classifications. These results indicate that reliable classifications depend not only on the model itself but also on the quality of the input images.

To further assess the effect of noise level on classification accuracy, we analyzed an additional simulated test data set with added white noise. The noise standard deviation σ_*N*_ was varied from 0.0 to 1.0 nm in 0.1 nm increments, with 0.8 million images generated for each noise level. We found that classification accuracy decreased with an increasing white noise level. Specifically, the exact-match accuracy at σ_*N*_ = 0.0, 0.2, 0.4, 0.6, 0.8, and 1.0 nm was 93.4%, 90.3%, 83.7%, 74.5%, 62.5%, and 47.7%, respectively (Figure S5B). Notably, allowing a tolerance of ±3 states (corresponding to an effective RMSD of 3.8 Å or lower) increased the accuracy to 99.9%, 99.8%, 99.4%, 98.3%, 95.7%, and 89.2% for the corresponding noise levels. Classification is likely to be hindered when strong noise overlaps with the key structural features of SecA. It is worth noting that the AE model was trained using noise levels in the range σ_*N*_ = 0.0–0.3 nm. Considering that the vertical resolution of HS-AFM experiments is typically 0.1 nm, noise levels as high as σ_*N*_ = 1.0 nm are unlikely under the experimental conditions. Nevertheless, the accuracy remained above 85% when a tolerance of ±3 states was allowed. These results indicate that DeepAFM is sufficiently robust to simple noise sources such as white noise.

### Other Factors Influencing Classification Performance

To understand other factors affecting classification performance in DeepAFM, we first examined how the extent of conformational sampling by MD simulations influences classification accuracy. We used the test images generated from the morphing snapshots (Figure 2B) and estimated the corresponding conformational states. For each snapshot, 100,000 images were generated by assigning random protein orientations, without adding white noise. Figure 4A shows the output probability distributions for Snapshots 1, 5, and 24. For Snapshot 1, state 1 or 2 (closed state) was assigned a combined average probability of 95.8%, indicating an accurate and confident classification. For Snapshot 24, state 18 (wide-open state) was assigned an average probability of 92.8%, also suggesting high accuracy and confidence. In contrast, for Snapshot 5, the output probabilities were broadly distributed. Major contributions arose from states 1, 2, 9, 12, and 13 (17.0%, 21.9%, 7.5%, 14.6%, and 13.9%, respectively), indicating substantial uncertainty with no single state being confidently assigned.

**Figure 4:**
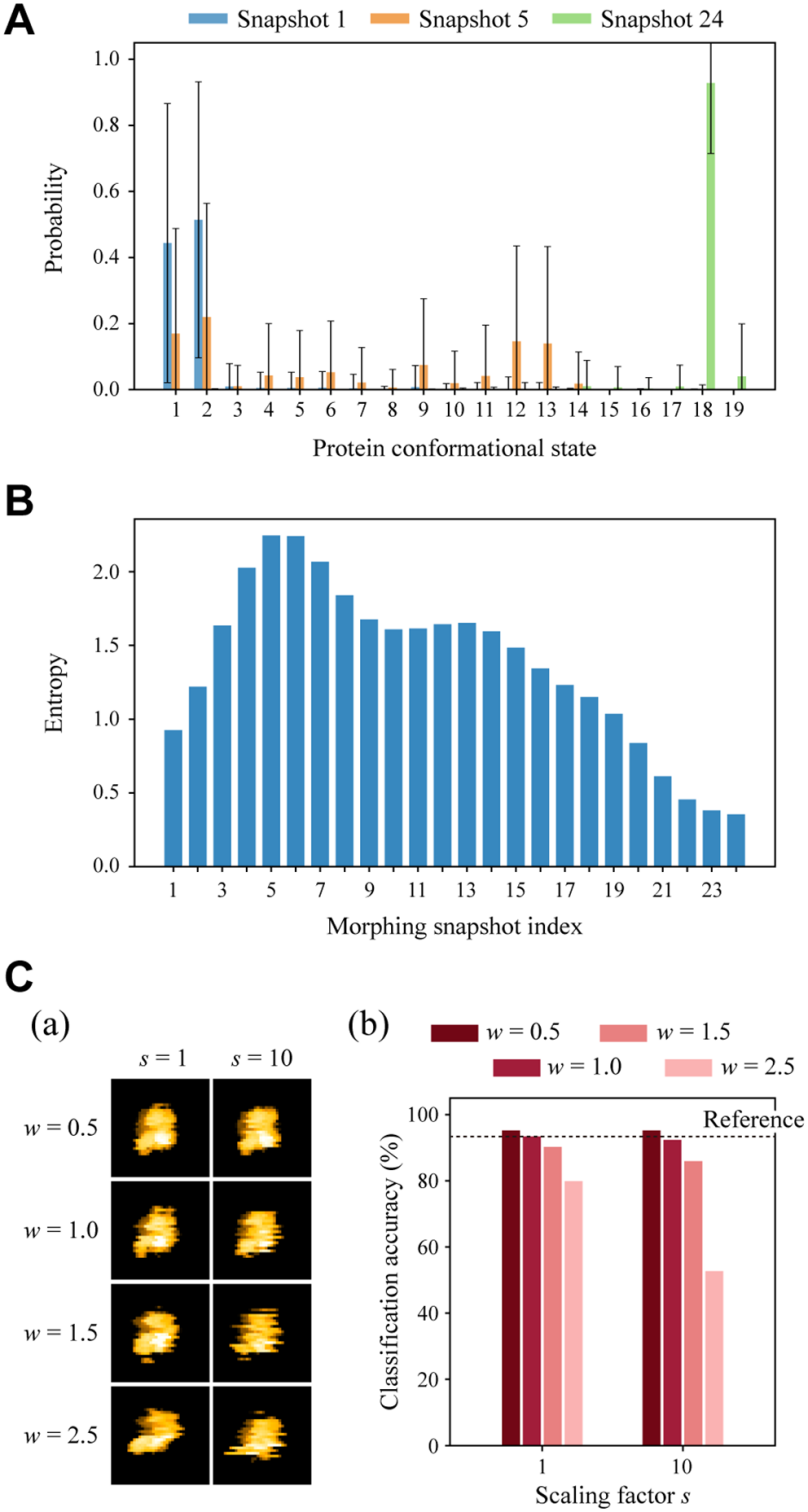
Performance of the trained AE on additional test data sets in relation to conformational sampling range and image distortions. (A) Probability distributions of conformational states for test images generated from Snapshots 1, 5, and 24 of the morphing trajectory, each averaged over 100,000 test images. Error bars represent standard deviations. (B) Entropy of the classified state distributions for Snapshots 1–24 along the morphing trajectory. (C) (a) Representative simulated images generated with different combinations of the scaling factors *s* and *w*. (b) Classification accuracy of conformational states for 100,000 test images generated for each parameter combination. The horizontal dashed line denotes the accuracy in the reference condition (*s* = 1, *w* = 1.0).

To quantify the classification uncertainty for each Snapshot, we computed the Shannon entropy $H\left(P\right)=-\sum_{i=1}^{n}p_{i}\ln p_{i}$ , where *P* = {*p*_1_, ..., *p*_*n*_} is the class probability distribution averaged over 100,000 test images per Snapshot, *p*_*i*_ is the average probability of the *i*-th conformational state, and *n* is the total number of states (*n* = 19). A low entropy value indicates that the classifications are concentrated on a particular state, suggesting a high confidence in the classification. As shown in Figure 4B, the entropy increases from Snapshots 1 to 5, then decreases from Snapshots 6 to 24. Notably, it sharply decreases for Snapshots 15–24. This trend aligns with the morphing trajectory moving away from the MD-sampled conformational space in the intermediate region and returning toward it later (Figure 2B). The entropy clearly correlated with the minimum RMSD between the MD trajectories and each morphing snapshot (Figure S6). These findings indicate that the classifier in the AE model becomes more confident when the protein depicted in the input image closely resembles conformations sampled in the MD ensemble. Therefore, sufficient sampling of relevant conformational states in MD simulations is essential for achieving accurate classification.

Next, we investigated how differences in distortion levels between input and training images affect the classification accuracy. To systematically evaluate the impact of distortion, we varied the two scaling factors, *s* and *w* (see above), when generating test images. First, we increased *s*, the scaling factor of the variance of the normal distributions used to generate Δ*x*, Δ*y*, Δϕ, Δθ, and Δψ. This increases the effective diffusion coefficient of the target protein. Second, we changed *w*, the factor applied to the corresponding maximum displacements (Δ*r*_max_, Δϕ_max_, Δθ_max_, and Δψ_max_) to control the spatial and angular ranges of motion. As illustrated in Figure 4C­(a), when the range of motion is small (i.e., low *w*), even rapid motion (high *s*) results in minimal distortion. In contrast, when *w* is large, increasing *s* leads to more pronounced image distortions. We generated 100,000 test images for each combination of *s* = 1 and 10 and *w* = 0.5, 1.0, 1.5, and 2.5 by randomly selecting MD snapshots and assigning protein orientations, without adding white noise. We evaluated classification accuracy using the AE trained under the reference conditions (*s* = 1, *w* = 1.0). Note that for *s* = 10, the parameter distributions become nearly uniform within the allowed range.

As shown in Figure 4C­(b), when *w* = 1.0, classification accuracy remained above 92.4% even as *s* increased to 10. When *w* = 0.5, the accuracy showed a slight improvement across all cases. In contrast, for *w* = 2.5, the accuracy declined markedly with increasing *s*, dropping to ∼50% at *s* = 10. This result indicates that classification accuracy is substantially reduced when the input distortion exceeds the range covered during training. Therefore, for reliable analysis of experimental data, the training data set must encompass a sufficiently broad range of distortions to capture the variability present in the input images.

### DeepAFM vs Rigid-Body Fitting

To further evaluate DeepAFM, we compared its classification accuracy with that of a conventional rigid-body fitting approach, specifically to evaluate its robustness against image distortion. Both methods were evaluated using distortion-added (*s* = 1, *w* = 1) and distortion-free (*s* = 0, *w* = 0) test images. For each condition, 10,000 test images were generated by randomly selecting MD snapshots and assigning protein orientations, without adding white noise. For DeepAFM, we employed the same AE model trained under the *s* = 1 and *w* = 1 condition. For rigid-body fitting, 19 cluster-center structures were used as reference models, and an exhaustive search was performed to identify the protein orientation and state that maximized the c.c. between the input and simulated images (see Materials and Methods for details).

We found that when only exact state matches were counted as correct, DeepAFM achieved 93.2% accuracy for distortion-free images and 91.4% accuracy for distortion-added images, with no misclassifications exceeding four states in either case (Figure S7A). In contrast, rigid-body fitting reached 63.9% accuracy for distortion-free images but dropped to 26.1% for distortion-added images, producing errors exceeding five states in 21 images and in 833 images, respectively (Figure S7B). For distortion-added images, when predictions within ±3 states were considered acceptable, DeepAFM achieved an accuracy of 99.8%, whereas rigid-body fitting achieved 79.4%. These results demonstrate that DeepAFM is robust to image distortion and noise, whereas rigid-body fitting is highly sensitive to such distortions.

Figure 5 presents two representative cases in which DeepAFM correctly classified the conformational state, whereas rigid-body fitting failed. In the DeepAFM results (Figure 5B), the denoised images closely resemble the corresponding input images, and the difference maps indicate that the larger discrepancies are mainly localized to noisy regions near the object boundaries. The 3D structures predicted from the denoised images (Figure 5B, right; see the figure caption or Materials and Methods for details) are consistent with the ground-truth 3D structures (Figure 5A, right). In the rigid-body fitting results (Figure 5C), we present the predicted 3D structures with the highest c.c. values and their corresponding simulated images. Although the simulated images closely resemble the input images, the difference maps reveal smaller discrepancies near the object boundaries (indicated by arrows) than those observed in the corresponding regions of the DeepAFM results. These regions correspond to the curved regions between NBD1 and HWD, as well as those between NBD1 and NBD2, which have similar shapes in both the wide-open and closed conformations. Because rigid-body fitting treats all pixels equally, the presence of strong noise in these regions can bias the fitting toward noise-dominated pixels, leading to poorer agreement around the PPXD domain (indicated by circles). Together, these results suggest that DeepAFM captures structural variations in key functional domains while minimizing the influence of noise during training, whereas rigid-body fitting can be more prone to overfitting structurally irrelevant regions.

**Figure 5:**
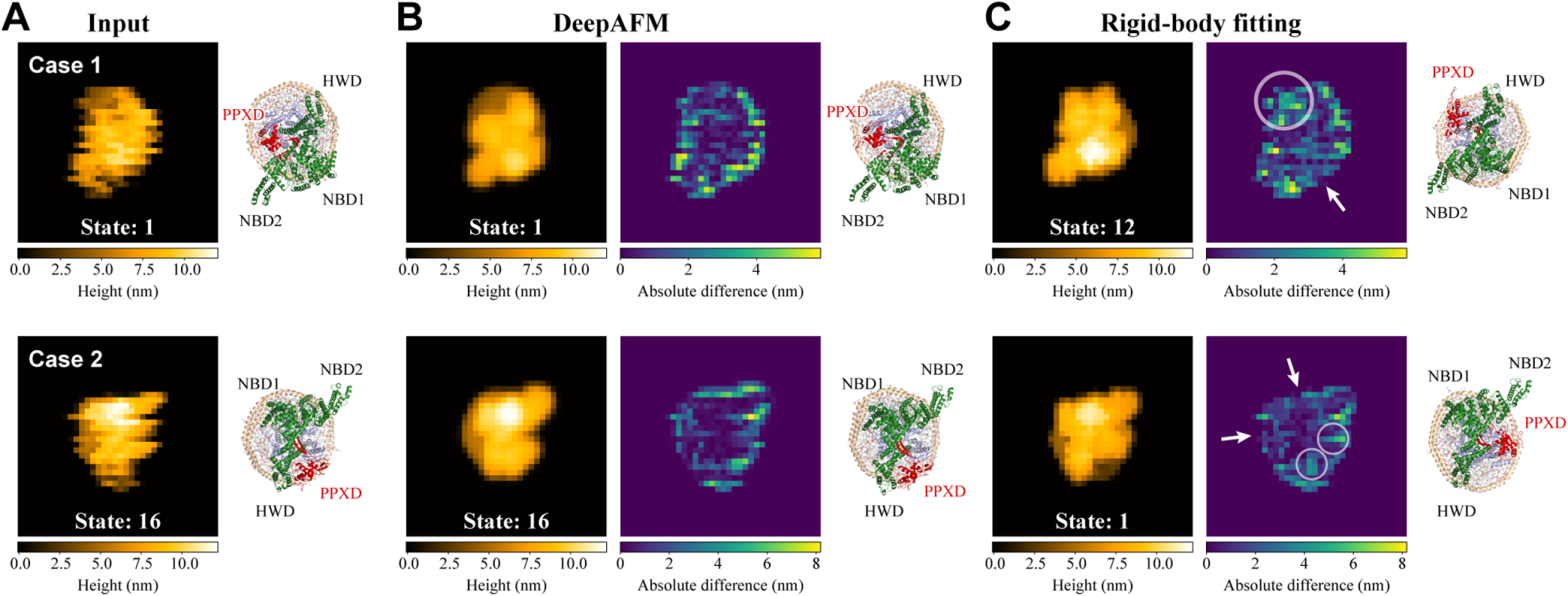
Representative results of DeepAFM and rigid-body fitting for distortion-added images. Examples are shown in which DeepAFM succeeded while rigid-body fitting failed. (A) Input test images and their corresponding ground-truth 3D structures. (B) From left to right: Denoised images obtained using DeepAFM, difference maps relative to the input images, and 3D structures obtained by rigid-body fitting of the cluster-center structure for each estimated conformational state onto the denoised image. (C) From left to right: Simulated images generated from the 3D structures with the highest c.c. values, difference maps relative to the input images, and corresponding 3D structures obtained by rigid-body fitting onto the input images.

### Application to the Experimental Images

Finally, we analyzed the experimental images of the SecYAEG–ND complex. A total of 18 images were examined using DeepAFM with the trained AE model and compared with the results from rigid-body fitting (see Materials and Methods for details). Figure 6 presents three representative examples (Exps 1–3), while the complete set of 18 images is summarized in Figure S8. DeepAFM effectively removes substantial noise from the experimental images while preserving the overall shape and size of the protein (compare Figure 6A,B, left). The locations of high-intensity regions are also well preserved in the denoised images. Based on the predicted 3D structures (Figure 6B, middle), features that are presumed to correspond to key structural elements are consistently found in the input and denoised images, including the groove between HWD and NBD1 (indicated by blue arrows), the groove between PPXD and NBD2 (green arrows), and the protruding NBD2 domain (magenta arrows). In addition, the attention maps suggest that the model predominantly focuses on regions that are likely to correspond to the PPXD domain (Figure 6B, right). Together, these observations indicate effective denoising for the experimental images. The conformational states of Exp 1 and Exp 3 were assigned to state 19, while Exp 2 was assigned to state 15. Since the input images of Exp 1 and Exp 2 capture the target in similar shapes and exhibit similar noise patterns, they were expected to be assigned to the same state. Although a difference of four states was output, the RMSD between their cluster centers is 3.3 Å, suggesting that the conformations in Exp 1 and Exp 2 are structurally similar.

**Figure 6:**
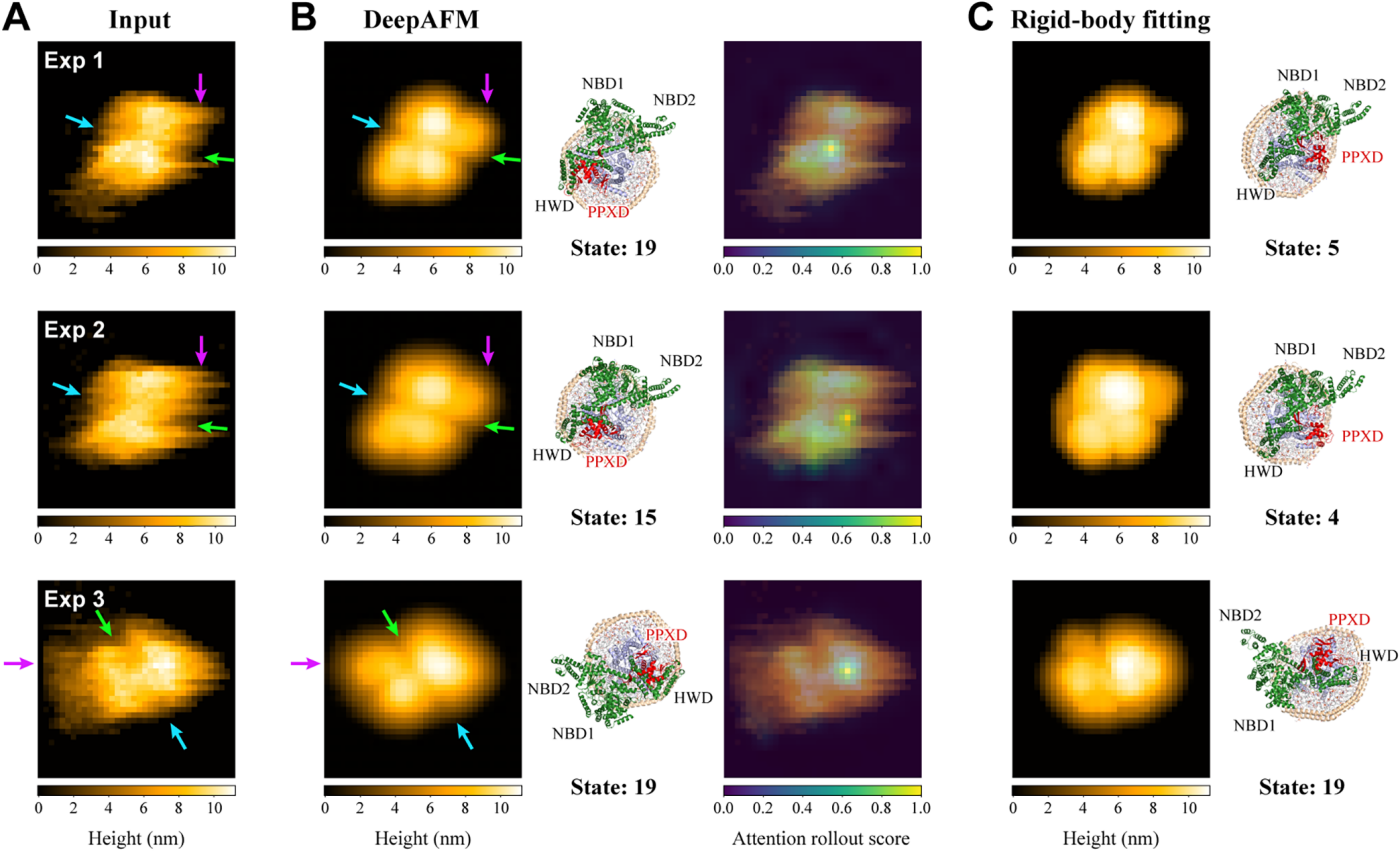
Comparison between DeepAFM and rigid-body fitting for three representative cases selected from the 18 experimental images. (A) Input experimental images. (B) From left to right: Denoised images obtained using DeepAFM, the corresponding 3D structures obtained by rigid-body fitting of the cluster-center structure for each estimated conformational state onto the denoised images, and attention rollout score overlaid on the input images. (C) Simulated images generated from the 3D structures with the highest c.c. values (left) and the corresponding 3D structures obtained by rigid-body fitting onto the input images (right).

Figure 6C presents the results of rigid-body fitting for Exps 1–3. Although the simulated AFM images closely resembled the input images, the conformational states assigned by rigid-body fitting partially differed from those assigned by DeepAFM. Specifically, Exps 1–3 were assigned to states 5, 4, and 19, respectively. Similar to DeepAFM, the rigid-body fitting assigned nearby states to Exp 1 and Exp 2. Notably, the predicted 3D structures (Figure 6C, right) exhibited a larger tilt relative to the AFM stage than those inferred from the denoised images (see Figure S9). From a physical standpoint, orientations in which the nanodisc is nearly parallel to the AFM stage are considered plausible, suggesting that the conformational states and inferred orientations in the DeepAFM results are likely to be more reliable. Given that rigid-body fitting is prone to overfitting in the presence of noise, performing rigid-body fitting after denoising and conformational state assignment, rather than directly on raw noisy experimental data, is expected to provide more accurate predictions of the corresponding 3D structures.

Figure 7 compares the distributions of conformational states obtained by using DeepAFM and rigid-body fitting for the 18 experimental images. DeepAFM assigned states 14, 15, 16, and 19, whereas rigid-body fitting yielded a much broader distribution, assigning states ranging from 1–11 as well as 18 and 19. In both methods, state 19 was assigned the highest probability. When the states are coarsely grouped into three conformational categories—closed (states 1–7), intermediate (states 8–12), and wide-open (states 13–19)—DeepAFM classified all 18 images as wide-open. In contrast, rigid-body fitting identified not only wide-open states but also closed and intermediate ones. Although the two methods yield different interpretations of the experimental data, the DeepAFM results are consistent with the previous experiments reported by Kanaoka et al.,^5^ in which only the wide-open state was observed in side-on HS-AFM measurements in the absence of a translocating preprotein. In addition, X-ray crystallography of *T. thermophilus* SecA without a translocating preprotein also indicates that the wide-open conformation is stable,^43^ suggesting that this conformation likely represents the resting state in this system. Considering that DeepAFM is more robust to noise than rigid-body fitting, its state assignments are likely to be more reliable. Our analysis of the end-up images further supports the conclusion that the wide-open state is predominant under these experimental conditions.

**Figure 7:**
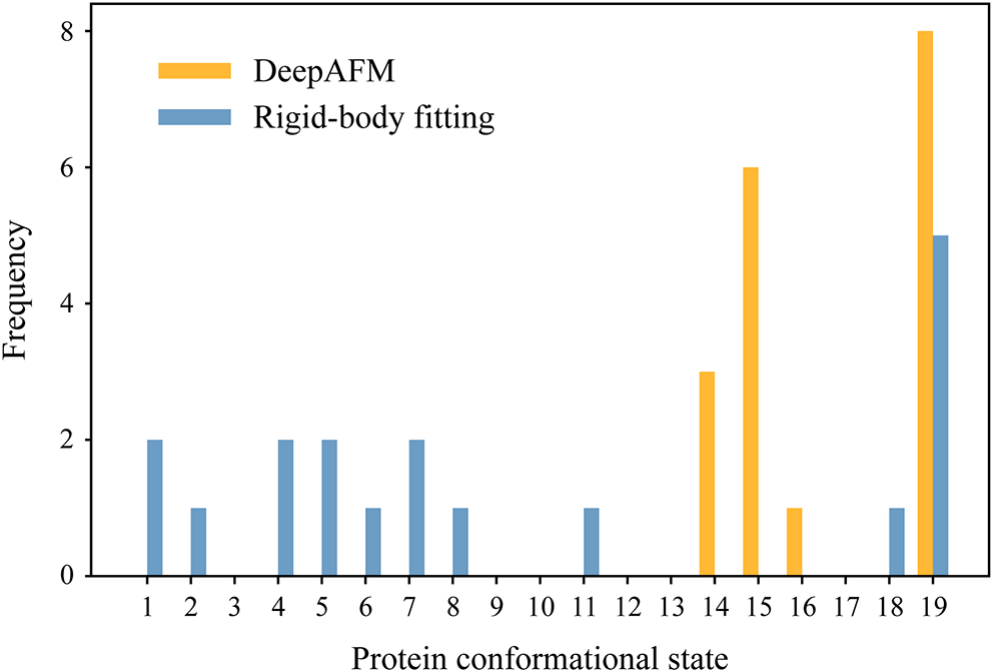
Distribution of protein conformational states identified by DeepAFM and rigid-body fitting across the 18 experimental images.

## Discussion

### Overview of DeepAFM

In this study, we developed DeepAFM, a novel algorithm that combines MD simulations with DL to denoise HS-AFM images and estimate protein conformational states. A key feature of DeepAFM is its use of training data comprising a large number of simulated images with realistic noise generated from MD snapshots. To better represent HS-AFM measurements, we introduced noise that mimics distortions arising from the Brownian motion of the target protein and temporal scanning lag. DeepAFM classified protein conformational states with over 90% accuracy on simulated test images with low white-noise levels and showed greater robustness to image distortions compared with conventional rigid-body fitting. When applied to experimental images of the SecYAEG–ND complex, DeepAFM effectively reduced the noise and produced results consistent with previous experimental observations. These findings suggest that DeepAFM offers a powerful tool for HS-AFM image analysis.

DeepAFM results should be interpreted with caution, as the training data are inherently synthetic, imposing intrinsic limitations on the analysis of the experimental images. Reliable interpretation of experimental HS-AFM images still requires comprehensive consideration of complementary analytical methods and independent experimental evidence. When DeepAFM successfully denoises images and rigid-body fitting yields simulated images that closely resemble the experimental images with consistent state assignments, the classification results can be regarded as highly reliable. Conversely, if DeepAFM fails to adequately denoise images or reproduce the overall protein shape, the reliability of conformational state assignments is likely reduced, and these cases may fall outside the range of analyzable data. Failures may arise from factors, including noise characteristics not fully captured by the model, insufficient conformational sampling in MD simulations, and unexpected conformational changes induced by mechanical perturbations from the AFM probe. Traditionally, computational analysis has relied on rigid-body and flexible fitting. In this context, DeepAFM provides a novel DL-assisted strategy that enables a more careful and integrative interpretation.

We expect that DeepAFM can be effectively extended to other systems through transfer learning. To evaluate this, we initialized the AE with weights pretrained on the SecYAEG–ND complex and applied it to two distinct targets: the magnesium ion transporter MgtE–nanodisc (ND) complex^32^ and the E6AP HECT catalytic domain.^44^ For both systems, we used simulated images. For MgtE–ND, we examined denoising performance, whereas for HECT, we assessed both denoising and conformational state classification. In MgtE–ND, transfer learning reduced the test MSE compared with training from scratch, indicating improved denoising (Figure S10). In HECT, it also reduced the test MSE and increased classification accuracy among three representative conformational states (Figure S11). These results suggest that features learned from SecYAEG–ND can be reused for structurally distinct targets. Nevertheless, because image characteristics and conformational landscapes are system-dependent, optimal performance generally requires retraining or fine-tuning for each case. Further systematic evaluation of transferability across diverse proteins will be important in future studies.

### Conformational Sampling

Our results demonstrate that sufficiently broad conformational sampling is essential for reliable denoising and accurate state estimation. Importantly, no universally optimal, system-independent strategy currently exists for sampling protein conformational transitions. When this framework is applied to a new system, the sampling strategy should be chosen according to the structural and dynamical properties of the target protein. Depending on the system, conformational diversity may be generated using approaches such as morphing-based interpolation,^42^ TMD,^40^ reduced-MSA subsampling in AlphaFold,^45^ NMA,^46^ or enhanced sampling techniques including replica-exchange MD (REMD),^47^ parallel cascade selection MD (PaCS-MD),^48^ and coarse-grained MD simulations.^49^ These methods can provide suitable initial conformations or pathways for subsequent MD refinement and a broader exploration of the conformational landscape.

As an application of the DeepAFM framework, we employed a TMD-based strategy for the SecYAEG–ND complex. Specifically, we first performed TMD simulations to generate an initial transition pathway, followed by multiple independent MD simulations to further explore the conformational space between the closed and wide-open states. SecA undergoes predominantly rigid-body motion during the conformational transition, suggesting a relatively simple conformational landscape. Although the sufficiency of conformational sampling cannot be rigorously assessed, AlphaFold 3 ^50^ produced intermediate structures in close proximity to the MD trajectories (Figure S12), providing independent support for the plausibility of the MD-sampled conformational space. These observations suggest that the TMD-based approach was reasonably effective at capturing the relevant conformational variability of the SecYAEG–ND complex.

For the generation of diverse conformations, reduced-MSA subsampling in AlphaFold 2 ^45^ could be an alternative approach. This strategy can produce multiple metastable conformations that may serve as starting structures for subsequent unbiased MD simulations to explore transitions among metastable states.^51^ Such ensembles may be particularly useful for systems lacking experimentally resolved intermediate structures. To explore this possibility, we applied reduced-MSA subsampling to the SecYAEG complex. However, the resulting models predominantly clustered around the closed-state conformation (Figure S12), and distinct open or wide-open states were not observed, possibly due to limitations of reduced-MSA subsampling or biases in available structural data. Although this approach did not successfully generate diverse conformations for SecYAEG, reduced-MSA subsampling remains a promising and computationally inexpensive strategy. When multiple metastable states are obtained, these structures could serve as useful seeds for MD simulations and for generating diverse training ensembles in the DeepAFM framework.

### Preparation of Training Data

In this study, 19 conformational states were defined by clustering the MD trajectories. This number was chosen to balance structural resolution and interpretability, while still representing the major conformational variations of SecA along the transition pathway and avoiding overpartitioning into redundant clusters. The resulting clusters exhibited an average within-cluster RMSD of ∼1.7 Å (Figure S3), indicating that each cluster represents a structurally similar conformational ensemble. For other systems, the optimal number of conformational states may vary, depending on the structural diversity observed in MD simulations or experimental data. As a practical guideline, clusters may be defined such that the RMSD within clusters remains below ∼2 Å, while the RMSD between clusters exceeds ∼3 Å, ensuring that each cluster corresponds to a structurally distinct conformational state.

Although cluster centers represent typical structures for each conformational state, we used MD snapshots rather than cluster centers themselves to train the DL model. DL models generally benefit from large and diverse training data sets, and relying solely on cluster centers would capture only limited structural variations within a state. Even when structures within a cluster are relatively similar, thermally sampled MD snapshots provide a broader set of structure–image pairs that reflect structural fluctuations within each state. This strategy maintains the robustness of the model across diverse systems and reduces the risk of missing potentially important conformations, particularly for proteins with substantial thermal fluctuations or high structural diversity.

The training data (noise-added images) must encompass the range of distortions observed in experimental images, which is achieved by incorporating a sufficient degree of Brownian motion of the target protein. We demonstrated that under these conditions, classification accuracy improves when the input images contain less distortion than those used for training (Figure 4C). The translational Brownian motion introduced in this study corresponds to an estimated diffusion coefficient of *D* = 6.3 × 10^–4^ μm^2^/s, assuming a pixel measurement time of 10 μs. For comparison, diffusion coefficients of lysozyme monomers adsorbed on mica have been reported as 9 × 10^–8^ μm^2^/s.^52^ In our experimental setup, SecY was tethered to the AFM stage—composed of a streptavidin 2D crystal—via two biotin linkers,^32^ likely restricting protein mobility relative to the previous system. Given the experimentally reported diffusion coefficients, the magnitude of Brownian motion introduced in this study is expected to be sufficient to analyze the experimental images of the SecYAEG–ND complex.

To model rotational Brownian motion, we parametrized the protein orientation using standard Euler angles. While this representation is straightforward, applying perturbations to the Euler angles does not correspond to isotropic rotational diffusion on *SO*(3). Here, rotational fluctuations were approximated by introducing small stochastic perturbations to the Euler angles. Although this approach does not strictly reproduce Brownian motion or the corresponding normal distribution on *SO*(3),^53^ the approximation is reasonable near a reference orientation. In our system, the nanodisc is oriented nearly parallel to the *XY* plane, restricting ϕ and θ to a narrow angular range. Because only small angular deviations are sampled, the bias inherent to Euler-angle parametrization is expected to have minimal impact in our system. More rigorous treatments of rotational fluctuations will be incorporated in future versions of DeepAFM to further improve the physical realism of the model.

### Evaluation Framework and Predictive Performance

DeepAFM is trained and validated using a random train–validation split within the MD-sampled conformational ensemble, where both training and validation data are drawn from the same set of clusters. This setup primarily evaluates the model’s ability to interpolate within the sampled conformational space rather than to predict entirely unseen regions. Because snapshots within the same cluster are often temporally correlated, strict statistical independence between the training and validation sets is not fully ensured, and the random split may therefore slightly overestimate performance. Although such temporal correlations can be mitigated using enhanced sampling methods, such as REMD, the evaluation still reflects interpolation within the sampled ensemble. Importantly, analysis of the morphing trajectory demonstrates that when input conformations lie outside the sampled ensemble, the model either assigns them to the closest known cluster or exhibits increased uncertainty. These observations indicate that the classification confidence depends on the proximity of input conformations to the MD-sampled ensemble. Because the true conformational state in experimental images is generally unknown, sufficiently broad sampling of relevant conformational states in MD simulations is essential for an accurate and confident classification.

In this study, we considered the deviation of the predicted class from the true class as a *post hoc* metric to quantify the classification performance. In typical classification tasks, the numerical difference between the predicted and true classes is usually not meaningful. The cross-entropy loss function does not explicitly penalize the magnitude of deviation between predicted and true class labels, and all misclassifications are treated equally regardless of any inherent ordering of class labels. In contrast, in our system, the conformational states correspond to a continuous structural transition with labels ordered from closed to wide-open conformations. Each state represents a distinct intermediate structure along this transition. In particular, a deviation of four or more states reflects a substantial conformational difference, corresponding to an effective RMSD greater than 4.4 Å (Figure S3). Consequently, unlike in typical classification tasks, the magnitude of deviation provides meaningful insight into the accuracy of the conformational state estimation. However, in systems with more complex energy landscapes, the relationship between state differences and structural changes may be less straightforward, and the magnitude of the state deviation should therefore be interpreted with caution.

### Computational Costs

Compared with conventional rigid-body and flexible fitting, DeepAFM requires substantial computational costs for MD simulations, image generation, and autoencoder training. These costs mainly depend on factors such as system size, conformational sampling method, and molecular model. For the SecYAEG–ND complex, all-atom MD simulations were performed to obtain a total trajectory of 2.1 μs (14,000 snapshots), typically taking more than one month on a cluster machine and ∼70 GB of disk storage. These costs can be reduced using coarse-grained models. The required size of the training data set is also system-dependent. For SecYAEG–ND, at least one million images were needed to reach 85% classification accuracy (Figure S13). Symmetric multimeric complexes such as F1-ATPase^33^ may require fewer images, as symmetry constrains conformational diversity. In contrast, intrinsically disordered proteins^54^ require broader conformational sampling in MD simulations and consequently larger training data sets. Furthermore, when the protein orientation is unknown, exhaustive angular sampling during image generation becomes necessary, substantially increasing both data set size and computational cost. Therefore, when DeepAFM is applied to other systems, careful consideration should be given to the structural complexity, conformational flexibility, and orientational variability of the target protein.

Since DeepAFM processes many simulated images, both file I/O and autoencoder training can become major computational bottlenecks. Existing tools for simulated AFM image generation, such as *afmize*,^55^ are not optimized for large-scale generation across wide parameter ranges or efficient data processing workflows. In this study, we developed an asynchronous parallel image-generation pipeline based on *ColabBTR* ^31^ using the Ray framework, in which ground-truth and noise-added image pairs are generated, chunked, and written directly to .tar archives. This pipeline achieved ∼0.5 million image pairs per hour on 128 CPU cores (2.0 GHz) and reduced storage for 8 million images to ∼130 GB. Autoencoder training on 8 million 36 × 36-pixel images required ∼64 h on two GPUs (e.g., RTX 2080 Ti). To efficiently process this large-scale data set, we employed WebDataset-based streaming to minimize memory overhead and enable scalable training. Once trained, the model classifies and denoises images within tens of milliseconds per image on a GPU.

### Comparison with Other Methods

To evaluate the reliability of the estimated protein conformational states, it is essential to compare the results not only with independent experimental data but also with those obtained from other computational approaches. Regarding the latter, this study specifically focused on a comparison with the conventional rigid-body fitting method. Comparisons with flexible-fitting methods^10–12^ are also important, but simple approaches require caution to avoid overfitting to noise, as they typically employ c.c. like rigid-body fitting. When flexible-fitting approaches are applied, algorithm-dependent limitations must also be considered. NMA-based methods^13^ typically rely on elastic network models, which reduce computational cost compared with all-atom MD simulations; however, certain systems, such as the lipid-containing nanodiscs employed in this study, may not be adequately described by a simple elastic network model. Particle filter methods^14^ are effective for analyzing structural dynamics in a time-resolved manner. However, they require the generation and evaluation of many replicas to identify conformations consistent with experimental data, leading to substantial computational cost. In practice, coarse-grained models are often necessary to make such calculations tractable. In this respect, DeepAFM is less constrained by these method-specific limitations. Recently, a data assimilation approach has been introduced,^15,16^ in which HS-AFM experimental data are integrated into coarse-grained MD simulations to better capture protein dynamics while explicitly accounting for measurement asynchrony, thereby reducing noise sensitivity. Since unambiguously determining true protein conformational states remains challenging for both DL-based and MD-based strategies, systematic cross-comparison among these methods will be crucial for gaining more robust insights into protein dynamics from experimental HS-AFM data.

## Summary

We present DeepAFM, an algorithm that integrates DL with MD simulations to estimate protein conformational states from HS-AFM images. By incorporating realistic noise arising from Brownian motion of the target protein and temporal line-scanning lag into the training data, DeepAFM shows stable performance across a range of image distortions. In addition to state estimation, the method enables noise reduction in experimental images, contributing to an improved interpretability. DeepAFM provides a new DL-assisted strategy for analyzing noisy HS-AFM data and facilitates studies of protein dynamics.

## Materials and Methods

### Neural Network Model

We designed our ViT-based multitask deep autoencoder (AE)^19^ with an asymmetric architecture (Figure S1 and Table S1), inspired by the asymmetric design of Masked Autoencoders.^56^ The AE comprises an encoder, a classifier, and a decoder, where the classifier assigns protein conformational states, and the decoder performs image denoising. The encoder is deeper and wider (12 layers, 512 dimensions) than the decoder (8 layers, 256 dimensions), encouraging the encoder to learn robust, high-level semantic representations rather than merely memorize low-level input details.

Given an input image *I* ( $I\in R^{H\times W}$ , where *H* and *W* denote the image height and width in pixels, respectively) and model parameters **w**, the AE outputs a denoised image $\hat{I^{*}\;}$ and a predicted protein conformational state *ŷ*. The mapping is expressed as $\left(\hat{I^{*}\;},ŷ\right)=Net\left(I;w\right)$ , where the asterisk denotes the denoised image and the hat indicates the network prediction. Here, *y* ∈ {1, ..., *N*} denotes a discrete conformational state defined through structural clustering, rather than a specific 3D atomic structure (e.g., Cartesian coordinates). To train the AE, we minimized the weighted loss function *L*(**w**) = α*L*_d_(**w**) + β*L*_c_(**w**), which combines the denoising loss *L*_d_(**w**) and the classification loss *L*_c_(**w**). The denoising loss *L*_d_ (**w**) was computed as the MSE between the ground-truth image I* and the predicted denoised image $\hat{I^{*}\;}$ : $L_{d}\left(w\right)=\frac{1}{N}\sum_{i=1}^{N}{\left(I_{i}^{*}-{\hat{I^{*}\;}}_{i}\right)}^{2}$ , where *N* is the number of training images. The classification loss *L*_c_(**w**) was defined as the cross-entropy between the ground-truth state *y* and the predicted state *ŷ*: $L_{c}\left(w\right)=-\sum_{k=1}^{K}y_{k}\log{ŷ}_{k}$ , where *K* is the number of states. To balance the loss scales, we set the weighting parameters to α = 100 and β = 1. The AE model was trained for 20 epochs in a distributed data parallel environment using two GPUs, with a total batch size of 192 (96 per GPU). The AdamW optimizer (weight decay = 0.0001) was used.^57^ For learning rate scheduling, the maximum learning rate was set to 0.002, and linear warmup was applied for the first five epochs,^58^ followed by cosine decay.^57^ The number of epochs was determined based on model performance on validation data during training. All DL models were implemented using PyTorch (v1.12.1) with a CUDA-enabled backend. Automatic mixed precision was employed to reduce memory consumption and accelerate training.

### Preparation of the Training and Testing Data Sets

Conformational sampling between the closed and wide-open states of the SecYAEG–ND complex was carried out using TMD^40^ and conventional MD simulations. The modeling protocol for the SecYAEG–ND complex, followed our previously reported method.^5^ We used the closed state of SecA as the initial structure for the simulation. The system size was 161 × 161 × 161 Å^3^, and the total number of atoms was ∼423,000. The CHARMM C36m force field parameters were used for proteins and lipids.^59,60^ First, energy minimization was performed for 10,000 steps, followed by equilibration for 25 ns in the *NVT* and *NPT* ensembles with positional restraints on the protein and lipids. Subsequently, to fully equilibrate the system, a 357 ns MD simulation was conducted in the *NPT* ensemble (*T* = 303.15 K, *P* = 1 atm) without positional restraints. The temperature and pressure were controlled using the Bussi thermostat and barostat,^61^ and the equations of motion were integrated with a time step of 3.5 fs, with the SHAKE algorithm and hydrogen mass repartitioning used for bond constraining.^62^ Nonbonded interactions were calculated with a cutoff distance of 12 Å, and long-range electrostatic interactions were computed using the particle-mesh Ewald method.^63^ Consistent with our previous study,^5^ the obtained SecA conformation remained in the closed state throughout the MD simulation.

The final snapshot was used as the initial structure for the TMD simulation. We conducted a 52.5 ns TMD simulation to induce the transition of SecA from the closed to the wide-open state, using the crystal structure of SecA in the wide-open state (PDB ID: 2IPC) as the reference coordinates. The TMD trajectory was divided evenly into 20 segments, and the first snapshot of each segment was selected as the initial structure for subsequent MD simulations. From these 20 structures, independent 105 ns MD simulations were conducted (2.1 μs in total) to sample intermediate conformations between the closed and wide-open states. PCA was applied to the combined trajectory data of the 20 independent simulations using only the frames from 31.5 to 105 ns (14,000 snapshots in total). Finally, clustering was performed by using the first six principal components. Here, a Gaussian mixture model was employed, with the number of clusters determined based on the Bayesian information criterion and the number of data points per cluster. For each cluster, the cluster-center structure was defined as the data point nearest to the cluster’s mean, calculated from all points assigned to that cluster. All simulations were performed using GENESIS ver. 2.1.1.^64^

The morphing calculations from the closed (AlphaFold2-predicted structure)^5^ to the wide-open state (PDB ID: 2IPC) were performed using the Morphit Pro server.^41^ Morphing was applied only to SecA. The resulting trajectory was then aligned to the SecA component of the SecYAEG–ND complex, using the initial structure of the TMD simulation as the reference, thereby generating a morphing trajectory for the entire complex.

### Generation of Simulated AFM Images

We randomly sampled structures with uniform probability from a total of 14,000 MD snapshots and generated simulated AFM images using our in-house program, which was partially based on *ColabBTR* developed by Matsunaga et al.^31^ To reduce computational cost during molecular surface scanning, only the protein Cα atoms were used, with each amino acid represented as a single sphere centered at the Cα position with an effective radius.^65^ For nanodisc lipids, only phosphorus atoms were considered, with radii taken from the PO4 beads in the MARTINI force field,^66^ while buried acyl chains were omitted. The following parameters were randomly varied when generating the simulated images. The AFM tip probe radius *R* was randomly selected from 1.0 to 3.0 nm in 0.1 nm increments, and the cone half-apex angle Θ from 5° to 30° in 1° increments. Under the experimental conditions, the nanodisc adopts an end-up orientation; therefore, it was aligned parallel to the *XY* plane. Accordingly, ϕ and θ were sampled from −20° to 20° in 1° increments, whereas ψ was sampled from −180° to 180° in 1° increments. Rotations were performed sequentially at the *z*-, *x*-, and *y*-axes (corresponding to ψ, ϕ, and θ, respectively). The stage height (*z* = 0) was defined as the minimum *z*-coordinate of the protein after the rotations. These randomly assigned protein lateral positions and orientations were denoted as (*x*_0_, *y*_0_, ϕ_0_, θ_0_, and ψ_0_). To generate noise-added images, the protein’s positions and orientations were further displaced from (*x*_0_, *y*_0_, ϕ_0_, θ_0_, and ψ_0_) to account for Brownian motion, as described in the Results section. During the data loading process of the training, the pixel values were randomly shifted in the *x* and *y* directions within ±3 pixels to randomize the protein’s lateral position within the image. In addition, random white noise was applied to the images at this stage. Gaussian white noise was added independently to each pixel as *I*(*x*,*y*) = *I*_0_(*x*,*y*) + η­(*x*,*y*), where *I*_0_(*x*,*y*) denotes the white-noise-free image intensity at pixel (*x*,*y*), and η­(*x*,*y*) ∼ *N*(0,σ_*N*_^2^), with σ_*N*_ controlling the noise magnitude. The image intensities of the training data were normalized by min–max scaling to stabilize model training, and the loss was computed using these normalized values. To convert the predicted intensities back to physical height units (nanometers), the decoder output was rescaled using the minimum and maximum heights of the corresponding input images expressed in nanometers.

### Rigid-Body Fitting to the Test, Denoised, and Experimental Images

Test images used for comparison between DeepAFM and rigid-body fitting comprised 10,000 images for both distortion-free and distortion-added cases. These images were generated from randomly selected MD snapshots with random protein orientations (ψ ranging from −180° to 180°, and ϕ and θ from −20° to 20°) and fixed AFM probe parameters (*R* = 1 nm, Θ = 10°). Rotations were performed sequentially at the *z*-, *x*-, and *y*-axes. No white noise was added.

For rigid-body fitting, we employed our own program to process large numbers of images. The program follows the same image-generation protocol and molecular rotation scheme used for generating the test images. An exhaustive grid-based search was performed, systematically sampling rotations and translations by using a standard Euler-angle rotation scheme. Because the nanodisc in our experimental setup is oriented nearly parallel to the *XY* plane, rotations around the *z*-axis were fully sampled, while rotations about the *x*- and *y*-axes were partially restricted. In contrast to globally uniform orientational sampling methods, such as Fibonacci-grid-based algorithms,^67,68^ our approach focuses on a local search around a reference orientation. Because only small angular deviations are explored, the nonuniformity associated with Euler-angle parametrization on *SO*(3) is expected to have minimal impact in our system. The similarity between reference and simulated images was evaluated using the c.c.,^55^ defined as

$$c.c.=\frac{\sum_{p}^{pixel}\left(I_{p}^{sim}-\left\langle I_{p}^{sim}\right\rangle \right)\left(I_{p}^{ref}-\left\langle I_{p}^{ref}\right\rangle \right)}{\sqrt{\sum_{p}^{pixel}{\left(I_{p}^{sim}-\left\langle I_{p}^{sim}\right\rangle \right)}^{2}}\sqrt{\sum_{p}^{pixel}{\left(I_{p}^{ref}-\left\langle I_{p}^{ref}\right\rangle \right)}^{2}}}$$

and the orientation yielding the highest c.c. was selected as the best-fit model.

In the rigid-body fitting for the test images (Figure 5C), 19 cluster-center structures were used as reference models. The exhaustive search was conducted with the following parameters: ψ ranging from −180° to 180° in 5° increments; ϕ and θ ranging from −30° to 30° in 5° increments; *R* = 1 nm; and Θ = 10°. In the rigid-body fitting for the denoised images derived from the test images (Figure 5B), the same protocol was applied, using the cluster-center structure corresponding to the assigned conformational state as the reference model. For the experimental data (Figure 6), *R* and Θ were additionally treated as grid parameters because the probe geometry is not known *a priori*. Increasing *R* or Θ decreases the effective spatial resolution of the simulated images, resulting in smoother topographical features and reduced structural detail.^34^ Consequently, the probe parameters can influence the identification of the best-fitting conformation. For both the denoised images derived from experimental data (Figure 6B) and the experimental images themselves (Figure 6C), the same rigid-body fitting procedure was employed, with *R* varied among 1.0, 2.0, and 3.0 nm, and Θ among 10°, 20°, and 30°. In all cases, rotations were applied sequentially to the *z*-, *x*-, and *y*-axes. Additionally, the lateral position of the protein was translated within ±3 pixels along both the *x* and *y* directions.

### Preprocessing of the Experimental AFM Images

The experimental details of HS-AFM imaging of the SecYAEG–ND complex have been reported previously by Haruyama et al.^32^ In these experiments, a two-dimensional streptavidin (SA) crystal was used as the AFM stage. Because the SA crystal surface is not perfectly flat and contains holes,^32^ the stage height was estimated as the average height of the lattice surface, excluding both the holes and the SecYAEG–ND complex. The holes were then filled with this average value, and the mean height was subtracted from the entire image. The protein was shifted to the center of the image. It was then cropped or zero-padded to 36 × 36 pixels to standardize the image size. When the image was used as input to the trained AE, the image intensities were expressed in physical height units (nanometers).

## Supplementary Material



## Data Availability

DeepAFM is publicly available as open-source software on GitHub (https://github.com/TUS-MoriLaboratory). The repository includes the full source code of the program, tutorials, and examples.

## Notes

Architecture of the ViT-based multitask deep autoencoder; hyperparameters of the ViT-based multitask deep autoencoder used in this study; comparison between experimental and simulated images of the SecYAEG–ND complex; RMSD between clusters; performance of the trained AE on the test images that contain no white noise; effect of white noise on prediction accuracy; minimum RMSD between MD trajectories and morphing snapshots vs entropy; effect of distortion on prediction accuracy; summary of the experimental data analysis; side views of denoised-image-derived and raw-image-derived 3D structures obtained by rigid-body fitting; application of transfer learning to the MgtE–ND complex and comparison with learning from scratch; application of transfer learning to the HECT domain and comparison with learning from scratch; PCA projection of *T. thermophilus* SecY–SecA structures; and comparison of model accuracy with training data sets of 0.1 million, 1 million, and 5 million images (PDF)

## References

[1] Ando T., Uchihashi T., Scheuring S. (2014). Filming biomolecular processes by
high-speed atomic force microscopy. Chem. Rev..

[2] Kodera N., Yamamoto D., Ishikawa R., Ando T. (2010). Video imaging of walking
myosin V by high-speed atomic force microscopy. Nature.

[3] Shibata M., Nishimasu H., Kodera N., Hirano S., Ando T., Uchihashi T., Nureki O. (2017). Real-space and real-time dynamics
of CRISPR-Cas9 visualized by high-speed atomic force microscopy. Nat. Commun..

[4] Sumino A., Sumikama T., Zhao Y., Flechsig H., Umeda K., Kodera N., Konno H., Hattori M., Shibata M. (2024). High-speed
atomic force microscopy reveals fluctuations and dimer splitting of
the N-terminal domain of GluA2 ionotropic glutamate receptor-auxiliary
subunit complex. ACS Nano.

[5] Kanaoka Y., Mori T., Nagaike W., Itaya S., Nonaka Y., Kohga H., Haruyama T., Sugano Y., Miyazaki R., Ichikawa M., Uchihashi T., Tsukazaki T. (2025). AFM observation
of protein translocation mediated by one unit of SecYEG-SecA complex. Nat. Commun..

[6] Umeda K., McArthur S. J., Kodera N. (2023). Spatiotemporal
resolution in high-speed
atomic force microscopy for studying biological macromolecules in
action. Microscopy.

[7] Scheuring S., Boudier T., Sturgis J. N. (2007). From high-resolution AFM topographs
to atomic models of supramolecular assemblies. J. Struct. Biol..

[8] Trinh M.-H., Odorico M., Pique M. E., Teulon J.-M., Roberts V. A., Ten Eyck L. F., Getzoff E. D., Parot P., Chen S. w., Pellequer J.-L. (2012). Computational
reconstruction of multidomain proteins
using atomic force microscopy data. Structure.

[9] Chaves R. C., Pellequer J.-L. (2013). DockAFM:
Benchmarking protein structures by docking
under AFM topographs. Bioinformatics.

[10] Niina T., Fuchigami S., Takada S. (2020). Flexible fitting of biomolecular
structures to atomic force microscopy images via biased molecular
simulations. J. Chem. Theory Comput..

[11] Wu X., Miyashita O., Tama F. (2024). Modeling conformational transitions
of biomolecules from atomic force microscopy images using normal mode
analysis. J. Phys. Chem. B.

[12] Amyot R., Miyashita O., Wu X., Takeda K., Kodera N., Konno H., Tama F., Flechsig H. (2025). Flexible fitting
to
infer atomistic-precision models of large-amplitude conformational
dynamics in biomolecules from high-speed atomic force microscopy imaging. ACS Nano.

[13] Vuillemot R., Pellequer J.-L., Grudinin S. (2025). Deciphering conformational dynamics
in AFM data using fast nonlinear NMA and FFT-based search with AFMFit. Commun. Biol..

[14] Fuchigami S., Niina T., Takada S. (2020). Particle filter
method to integrate
high-speed atomic force microscopy measurements with biomolecular
simulations. J. Chem. Theory Comput..

[15] Kubo S., Kato S., Nakamura K., Kodera N., Takada S. (2020). Resolving
the data asynchronicity in high-speed atomic force microscopy measurement
via the Kalman Smoother. Sci. Rep..

[16] Kato S., Takada S., Fuchigami S. (2023). Particle smoother
to assimilate asynchronous
movie data of high-speed AFM with MD simulations. J. Chem. Theory Comput..

[17] He, K. ; Zhang, X. ; Ren, S. ; Sun, J. Deep residual learning for image recognition. In Proceedings of the IEEE Conference on Computer Vision and Pattern Recognition (CVPR 2016); IEEE, 2016; pp 770–778.

[18] Krizhevsky, A. ; Sutskever, I. ; Hinton, G. E. ImageNet classification with deep convolutional neural networks. In Advances in Neural Information Processing Systems; Curran Associates, Inc., 2012.

[19] Dosovitskiy A., Beyer L., Kolesnikov A., Weissenborn D., Zhai X., Unterthiner T., Dehghani M., Minderer M., Heigold G., Gelly S., Uszkoreit J., Houlsby N. (2020). An image is worth 16 × 16 words:
Transformers
for image recognition at scale. arXiv.

[20] Ronneberger, O. ; Fischer, P. ; Brox, T. U-Net: Convolutional networks for biomedical image segmentation. In Medical Image Computing and Computer-Assisted Intervention–Miccai 2015:18th International Conference; Springer, 2015; pp 234–241.

[21] Zhang K., Zuo W., Chen Y., Meng D., Zhang L. (2017). Beyond a Gaussian Denoiser:
Residual learning of deep CNN for image denoising. IEEE Trans. Image Process..

[22] Giri N., Roy R. S., Cheng J. (2023). Deep learning for reconstructing
protein structures from cryo-EM density maps: Recent advances and
future directions. Curr. Opin. Struct. Biol..

[23] Belthangady C., Royer L. A. (2019). Applications, promises, and pitfalls
of deep learning
for fluorescence image reconstruction. Nat.
Methods.

[24] Masud N., Rade J., Hasib M. H. H., Krishnamurthy A., Sarkar A. (2024). Machine learning approaches
for improving atomic force
microscopy instrumentation and data analytics. Front. Phys..

[25] Sokolov I. (2024). On machine
learning analysis of atomic force microscopy images for image classification,
sample surface recognition. Phys. Chem. Chem.
Phys..

[26] Carracedo-Cosme J., Romero-Muñiz C., Pérez R. (2021). A deep learning approach for molecular
classification based on AFM images. Nanomaterials.

[27] Kim Y.-J., Lim J., Kim D.-N. (2022). Accelerating AFM characterization via deep-learning-based
image super-resolution. Small.

[28] Park J., Cheong D. Y., Lee G., Han C. E. (2025). Deep learning-based
denoising for unbiased analysis of morphology and stiffness in amyloid
fibrils. Comput. Biol. Med..

[29] Ziatdinov M., Zhang S., Dollar O., Pfaendtner J., Mundy C. J., Li X., Pyles H., Baker D., De Yoreo J. J., Kalinin S. V. (2021). Quantifying the dynamics of protein
self-organization using deep learning analysis of atomic force microscopy
data. Nano Lett..

[30] Degenhardt M. F. S., Degenhardt H. F., Bhandari Y. R., Lee Y.-T., Ding J., Yu P., Heinz W. F., Stagno J. R., Schwieters C. D., Watts N. R., Wingfield P. T., Rein A., Zhang J., Wang Y.-X. (2025). Determining structures
of RNA conformers using AFM and deep neural networks. Nature.

[31] Matsunaga Y., Fuchigami S., Ogane T., Takada S. (2023). End-to-end differentiable
blind tip reconstruction for noisy atomic force microscopy images. Sci. Rep..

[32] Haruyama T., Sugano Y., Kodera N., Uchihashi T., Ando T., Tanaka Y., Konno H., Tsukazaki T. (2019). Single-unit
imaging of membrane protein-embedded nanodiscs from two oriented sides
by high-speed atomic force microscopy. Structure.

[33] Uchihashi T., Iino R., Ando T., Noji H. (2011). High-speed atomic force
microscopy reveals rotary catalysis of rotorless F_1_-ATPase. Science.

[34] Amyot R., Flechsig H. (2020). BioAFMviewer: An interactive
interface for simulated
AFM scanning of biomolecular structures and dynamics. PLoS Comput. Biol..

[35] Tanaka Y., Sugano Y., Takemoto M., Mori T., Furukawa A., Kusakizako T., Kumazaki K., Kashima A., Ishitani R., Sugita Y., Nureki O., Tsukazaki T. (2015). Crystal structures
of SecYEG in lipidic cubic phase elucidate a precise resting and a
peptide-bound state. Cell Rep..

[36] Rapoport T. A., Li L., Park E. (2017). Structural
and mechanistic insights into protein translocation. Annu. Rev. Cell Dev. Biol..

[37] Sugano Y., Furukawa A., Nureki O., Tanaka Y., Tsukazaki T. (2017). SecY-SecA
fusion protein retains the ability to mediate protein transport. PLoS One.

[38] Osborne A. R., Clemons W. M., Rapoport T. A. (2004). A large conformational
change of the translocation ATPase SecA. Proc.
Natl. Acad. Sci. U.S.A..

[39] Smets D., Loos M. S., Karamanou S., Economou A. (2019). Protein transport across
the bacterial plasma membrane by the Sec pathway. Protein J..

[40] Schlitter J., Engels M., Krüger P., Jacoby E., Wollmer A. (1993). Targeted molecular
dynamics simulation of conformational change-Application to the T
↔ R transition in insulin. Mol. Simul..

[41] Veevers R., Hayward S. (2018). Morphing and docking
visualisation of biomolecular
structures using multi-dimensional scaling. J. Mol. Graphics Modell..

[42] Weiss, D. R. ; Koehl, P. Morphing methods to visualize coarse-grained protein dynamics. In Protein Dynamics: Methods and Protocols; Springer, 2014; pp 271–282. 10.1007/978-1-62703-658-0_15 24061927

[43] Vassylyev D. G., Mori H., Vassylyeva M. N., Tsukazaki T., Kimura Y., Tahirov T. H., Ito K. (2006). Crystal Structure of
the Translocation ATPase SecA from Thermus thermophilus Reveals a
Parallel, Head-to-Head Dimer. J. Mol. Biol..

[44] Takeda K., Flechsig H., Muro I., Amyot R., Kobayashi F., Kodera N., Ando T., Konno H. (2023). Structural dynamics
of E6AP E3 ligase HECT domain and involvement of a flexible hinge
loop in the ubiquitin chain synthesis mechanism. Nano Lett..

[45] del
Alamo D., Sala D., McHaourab H. S., Meiler J. (2022). Sampling alternative conformational states of transporters
and receptors with AlphaFold2. eLife.

[46] Bahar I., Rader A. J. (2005). Coarse-grained normal mode analysis
in structural biology. Curr. Opin. Struct. Biol..

[47] Sugita Y., Okamoto Y. (1999). Replica-exchange molecular
dynamics method for protein
folding. Chem. Phys. Lett..

[48] Harada R., Kitao A. (2013). Parallel cascade selection molecular
dynamics (PaCS-MD) to generate
conformational transition pathway. J. Chem.
Phys..

[49] Li W., Wang W., Takada S. (2014). Energy landscape views for interplays
among folding, binding, and allostery of calmodulin domains. Proc. Natl. Acad. Sci. U.S.A..

[50] Abramson J., Adler J., Dunger J., Evans R., Green T., Pritzel A., Ronneberger O., Willmore L., Ballard A. J., Bambrick J., Bodenstein S. W., Evans D. A., Hung C.-C., O’Neill M., Reiman D., Tunyasuvunakool K., Wu Z., Žemgulytė A., Arvaniti E., Beattie C., Bertolli O., Bridgland A., Cherepanov A., Congreve M., Cowen-Rivers A. I., Cowie A., Figurnov M., Fuchs F. B., Gladman H., Jain R., Khan Y. A., Low C. M. R., Perlin K., Potapenko A., Savy P., Singh S., Stecula A., Thillaisundaram A., Tong C., Yakneen S., Zhong E. D., Zielinski M., Žídek A., Bapst V., Kohli P., Jaderberg M., Hassabis D., Jumper J. M. (2024). Accurate structure prediction of
biomolecular interactions with AlphaFold 3. Nature.

[51] Meller A., Bhakat S., Solieva S., Bowman G. R. (2023). Accelerating cryptic
pocket discovery using AlphaFold. J. Chem. Theory
Comput..

[52] Mulheran P. A., Pellenc D., Bennett R. A., Green R. J., Sperrin M. (2008). Mechanisms
and dynamics of protein clustering on a solid surface. Phys. Rev. Lett..

[53] Nikolayev D. I., Savyolov T. I. (1997). Normal distribution
on the rotation group SO(3). Texture, Stress,
Microstruct..

[54] Yamamoto H., Fujioka Y., Suzuki S. W., Noshiro D., Suzuki H., Kondo-Kakuta C., Kimura Y., Hirano H., Ando T., Noda N. N., Ohsumi Y. (2016). The intrinsically disordered protein
Atg13 mediates supramolecular assembly of autophagy initiation complexes. Dev. Cell.

[55] Niina T., Matsunaga Y., Takada S. (2021). Rigid-body fitting to atomic force
microscopy images for inferring probe shape and biomolecular structure. PLoS Comput. Biol..

[56] He K., Chen X., Xie S., Li Y., Dollár P., Girshick R. (2021). Masked autoencoders
are scalable vision learners. arXiv.

[57] Loshchilov I., Hutter F. (2019). Decoupled weight decay regularization. arXiv.

[58] Goyal P., Dollár P., Girshick R., Noordhuis P., Wesolowski L., Kyrola A., Tulloch A., Jia Y., He K. (2018). Accurate, large minibatch SGD: Training ImageNet in 1 h. arXiv.

[59] Klauda J. B., Venable R. M., Freites J. A., O’Connor J. W., Tobias D. J., Mondragon-Ramirez C., Vorobyov I., MacKerell A. D., Pastor R. W. (2010). Update of the CHARMM all-atom additive
force field for lipids: Validation on six lipid types. J. Phys. Chem. B.

[60] Huang J., Rauscher S., Nawrocki G., Ran T., Feig M., de Groot B. L., Grubmuller H., MacKerell A. D. (2017). CHARMM36m: An improved force field
for folded and intrinsically
disordered proteins. Nat. Methods.

[61] Bussi G., Donadio D., Parrinello M. (2007). Canonical sampling through velocity
rescaling. J. Chem. Phys..

[62] Ryckaert J.-P., Ciccotti G., Berendsen H. J. C. (1977). Numerical
integration of the cartesian
equations of motion of a system with constraints: molecular dynamics
of *n*-alkanes. J. Comput. Phys..

[63] Essmann U., Perera L., Berkowitz M. L., Darden T., Lee H., Pedersen L. G. (1995). A smooth particle
mesh Ewald method. J. Chem. Phys..

[64] Jung J., Yagi K., Tan C., Oshima H., Mori T., Yu I., Matsunaga Y., Kobayashi C., Ito S., Ugarte La
Torre D., Sugita Y. (2024). GENESIS 2.1: High-performance molecular
dynamics software for enhanced sampling and free-energy calculations
for atomistic, coarse-grained, and quantum mechanics/molecular mechanics
models. J. Phys. Chem. B.

[65] Kim Y. C., Hummer G. (2008). Coarse-grained models for simulations
of multiprotein
complexes: Application to ubiquitin binding. J. Mol. Biol..

[66] Souza P. C. T., Alessandri R., Barnoud J., Thallmair S., Faustino I., Grünewald F., Patmanidis I., Abdizadeh H., Bruininks B. M. H., Wassenaar T. A., Kroon P. C., Melcr J., Nieto V., Corradi V., Khan H. M., Domański J., Javanainen M., Martinez-Seara H., Reuter N., Best R. B., Vattulainen I., Monticelli L., Periole X., Tieleman D. P., de Vries A. H., Marrink S. J. (2021). Martini 3: A general purpose force
field for coarse-grained
molecular dynamics. Nat. Methods.

[67] Swinbank R., James Purser R. (2006). Fibonacci grids: A novel approach
to global modelling. Q. J. R. Meteorol. Soc..

[68] González Á. (2010). Measurement
of areas on a sphere using Fibonacci and Latitude–Longitude
lattices. Math. Geosci..

