# Supplementary material for: Estimating Protein Conformational States from High-Speed AFM Images with Molecular Dynamics and Deep Learning
Source: J Chem Inf Model. 2026 Apr 4;66(8):4484–98. doi: 10.1021/acs.jcim.6c00142 (PMC13126631; doi:10.1021/acs.jcim.6c00142)
Supplement: Supplementary file 1 [file ci6c00142_si_001.pdf]

Supporting Information

Estimating Protein Conformational States from High-Speed AFM Images with Molecular Dynamics and Deep Learning

Katsuki Sato[1], Yui Kanaoka[2], Tomoya Tsukazaki[3],
Takayuki Uchihashi[2, 4, 5, 6], and Takaharu Mori[1,*]

[1] Department of Chemistry, Faculty of Science, Tokyo University of Science, Shinjuku-ku, Tokyo 162-8601, Japan
[2] Department of Physics, Graduate School of Science, Nagoya University, Furo-cho, Chikusa-ku, Nagoya, Aichi 464-8602, Japan
[3] Nara Institute of Science and Technology, Ikoma, Nara 630-0192, Japan
[4] Exploratory Research Center on Life and Living Systems (ExCELLS), National Institutes of Natural Sciences, 5-1 Higashiyama, Myodaiji, Okazaki 444-8787, Aichi, Japan
[5] Institute for Glyco-Core Research (IGCORE), Nagoya University, Furo-Cho, Chikusa-Ku, Nagoya 464-8601, Aichi, Japan
[6] Quantum-Based Frontier Research Hub for Industry Development (Q-BReD), Nagoya University, Nagoya 464-8601, Japan

Corresponding author:

* Takaharu Mori, Department of Chemistry, Faculty of Science, Tokyo University of Science
e-mail: t.mori@rs.tus.ac.jp

List of contents

Figure S1. Architecture of the ViT-based multi-task deep autoencoder

Table S1. Hyperparameters of the ViT-based multi-task deep autoencoder used in this study

Figure S2. Comparison between experimental and simulated images of the SecYAEG–ND complex

Figure S3. RMSD between clusters

Figure S4. Performance of the trained AE on the test images that contain no white noise

Figure S5. Effect of white noise on prediction accuracy

Figure S6. Minimum RMSD between MD trajectories and morphing snapshots vs. entropy.

Figure S7. Effect of distortion on prediction accuracy

Figure S8. Summary of the experimental data analysis

Figure S9. Side views of denoised-image–derived and raw-image–derived 3D structures obtained by rigid-body fitting

Figure S10. Application of transfer learning to the MgtE–ND complex and comparison with learning from scratch

Figure S11. Application of transfer learning to the HECT domain and comparison with learning from scratch

Figure S12. PCA projection of *T. thermophilus* SecY–SecA structures

Figure S13. Comparison of model accuracy with training datasets of 0.1 million, 1 million, and 5 million images

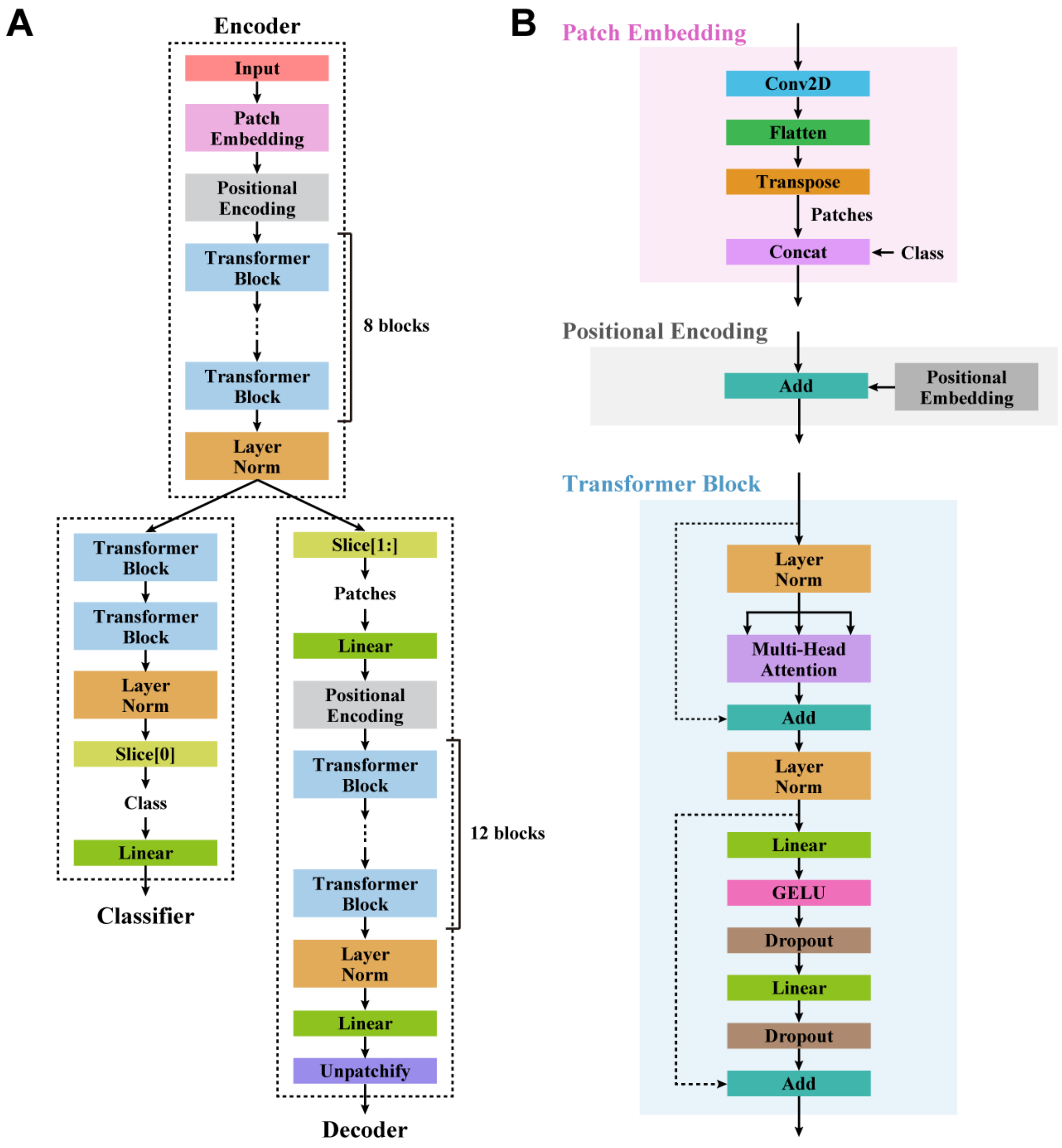

Figure S1. Architecture of the ViT-based multi-task deep autoencoder. (A) Overview of the architecture. The encoder output is divided into two branches: the classifier receives all tokens and uses only the class token before the final linear layer for protein state prediction, while the decoder processes patch tokens for image denoising and reconstructs the image via an unpatchify operation. (B) Detailed schematics of the patch embedding, positional encoding, and Transformer block. Patch embedding uses a 2D convolution to project image patches into embedding vectors, followed by prepending a class token. Learnable positional embeddings initialized with a truncated normal distribution are added to all tokens. Each Transformer block consists of multi-head self-attention and a feed-forward network with GELU activation, with residual connections after each sublayer.

Table S1. Hyperparameters of the ViT-based multi-task deep autoencoder used in this study.

Module	Parameter	Value
Global Settings	Input Size	36×36×1
	Patch Size	3×3
	Activation Function	GELU
	Dropout	0.1
Encoder (ViT)	Patch Embedding	Conv2d (Kernel=3, Stride=3)
	Positional Encoding	Learnable Parameters
	Input Tokens	Patches + Class Token
	Embedding Dimension	512
	Depth	12 layers
	Attention Heads	8 heads
	MLP Ratio	4
Decoder (ViT)	Input Tokens	Encoded Patches
	Input Projection	Linear (512 to 256 dim)
	Positional Encoding	Learnable Parameters
	Embedding Dimension	256
	Depth	8 layers
	Attention Heads	4 heads
	MLP Ratio	4
	Final Projection	Linear (to Patch Pixels)
Classifier (ViT)	Input Tokens	All Encoded Tokens
	Embedding Dimension	512
	Depth	2 layers
	Attention Heads	8 heads
	MLP Ratio	4
	Final Projection	Linear (to 19 Classes)

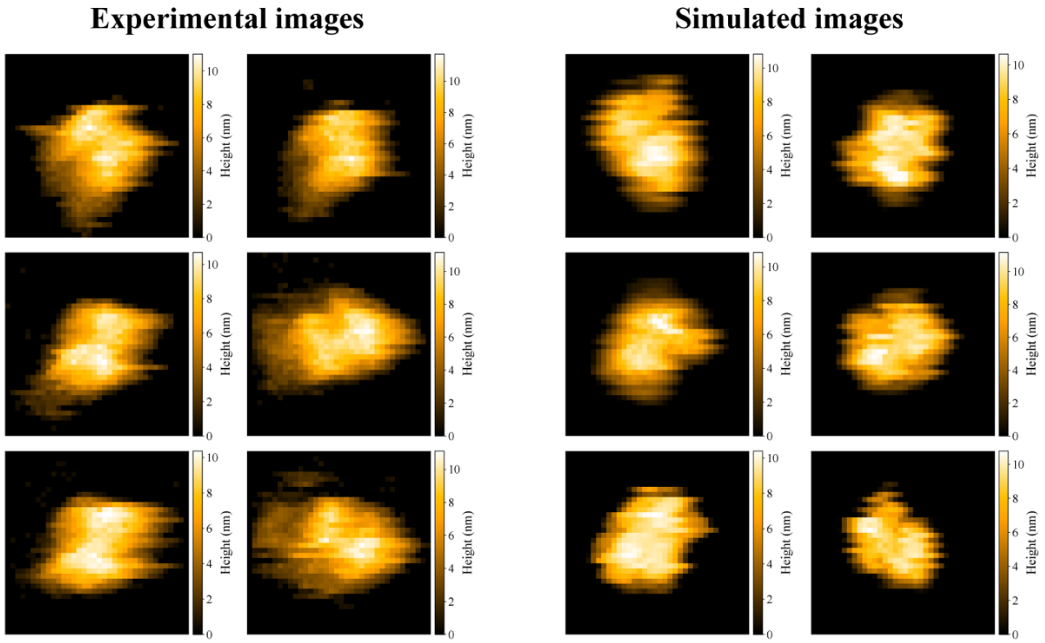

Figure S2. Comparison between experimental and simulated images of the SecYAEG–ND complex. (Left) Representative experimental images. (Right) Noise-added images generated from the 3D structure under the same conditions as the training data. These images were chosen for their close resemblance to the experimental ones in both noise pattern and overall molecular shape.

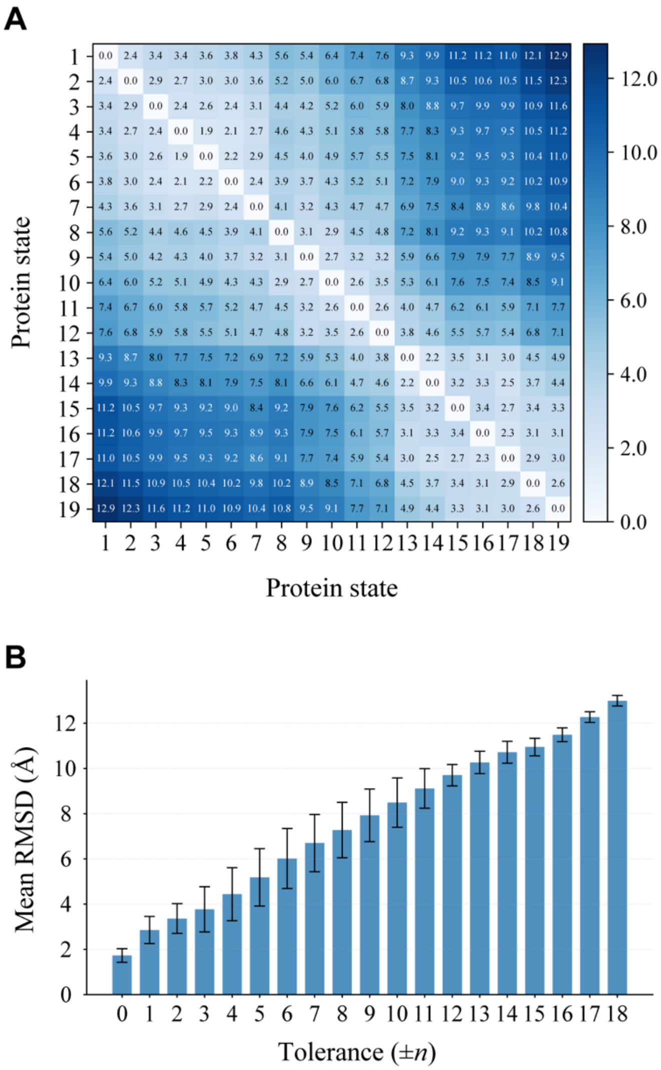

Figure S3. RMSD between clusters. (A) RMSD between cluster-center structures. (B) Mean RMSD between each cluster center and structures belonging either to the same cluster or to different clusters. For a given reference cluster i, RMSDs were calculated between the cluster center of cluster i and structures in cluster $i \pm n$. The horizontal axis represents the cluster index offset (n). Error bars indicate the minimum and maximum RMSD values.

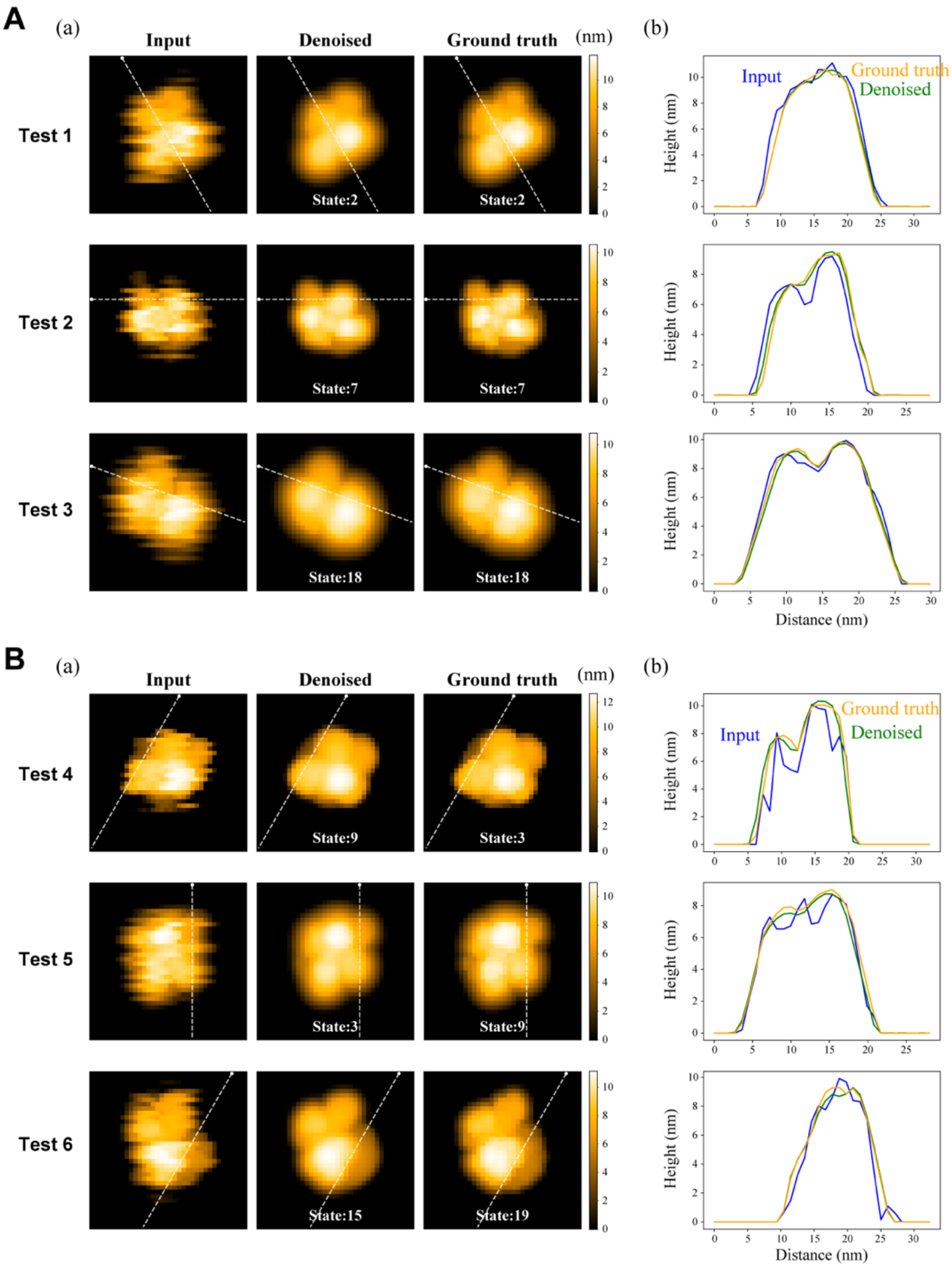

Figure S4. Performance of the trained AE on the test images that contain no white noise. (A) Successful examples and (B) unsuccessful examples. (a) From left to right: input, denoised, and ground-truth images. (b) Height profiles extracted along the dotted lines shown in (a).

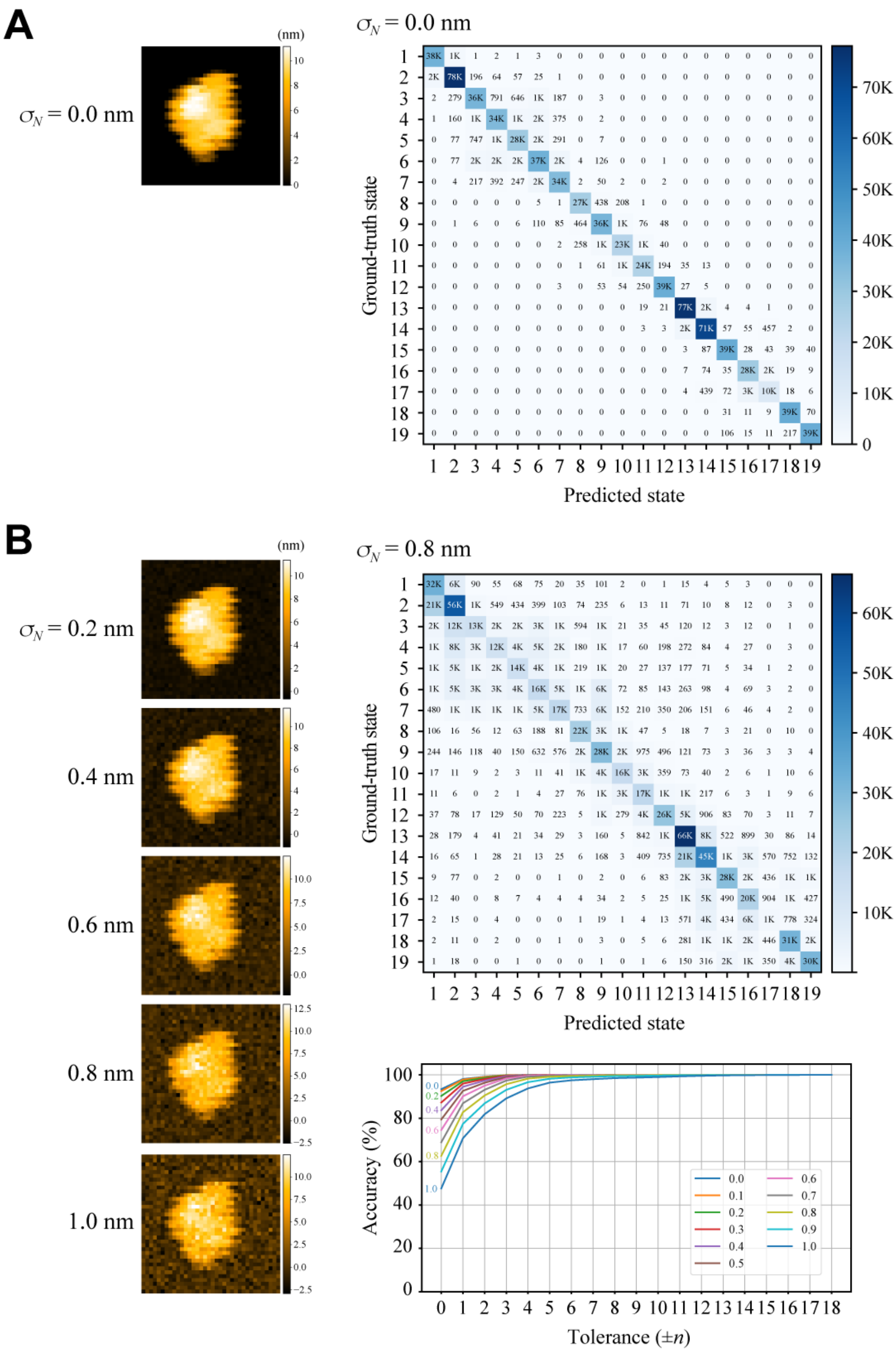

Figure S5. Effect of white noise on prediction accuracy. (A) Representative image (left) and confusion matrix between the predicted and ground-truth states for σ_N = 0.0 nm (right). (B) Representative images for σ_N = 0.2, 0.4, 0.6, 0.8, and 1.0 nm (left), confusion matrix for σ_N = 0.8 nm (top right), and prediction accuracy as a function of tolerance for σ_N = 0.1–1.0 nm (bottom right). In the confusion matrices, values greater than 1,000 are indicated using K (K = 1,000).

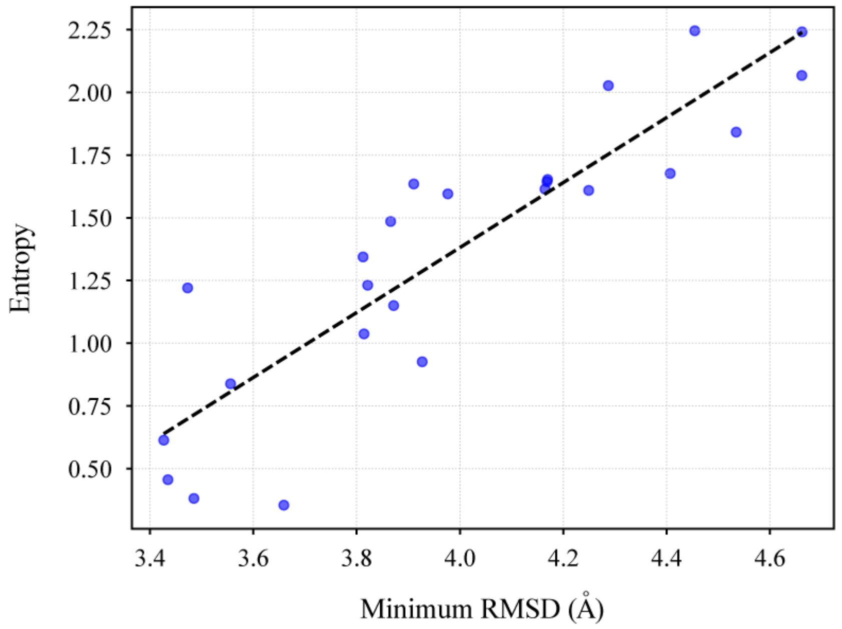

Figure S6. Minimum RMSD between MD trajectories and morphing snapshots vs. entropy. The RMSD was calculated using the Cα atoms of SecA. The correlation coefficient was $R^2 = 0.8882$.

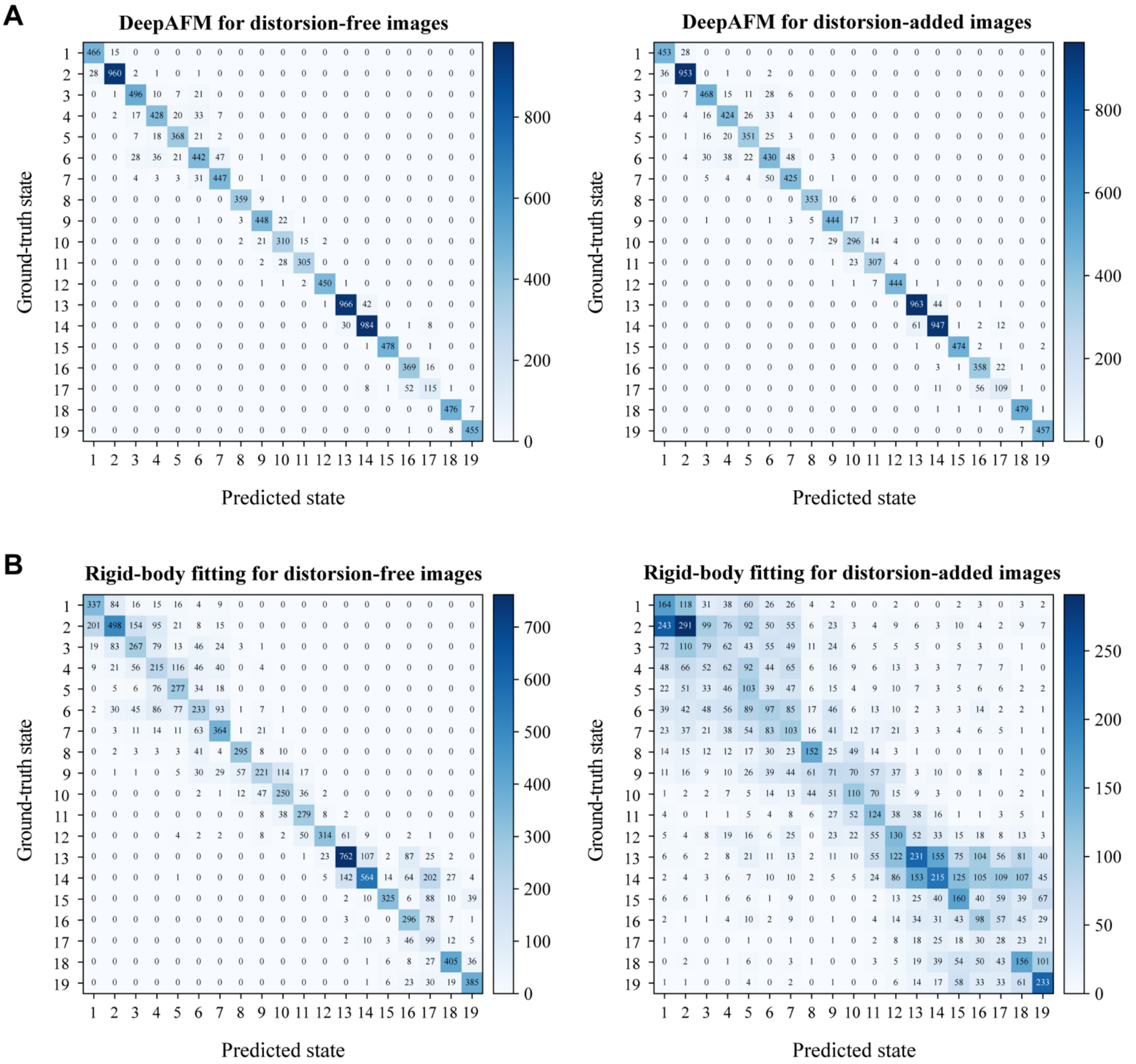

Figure S7. Effect of distortion on prediction accuracy. Confusion matrices between the predicted and ground-truth states for (A) DeepAFM for distortion-free images (left) and for distortion-added images (right), and (B) rigid-body fitting for distortion-free images (left) and for distortion-added images (right). In the confusion matrices, values greater than 1,000 are indicated using K (K = 1,000).

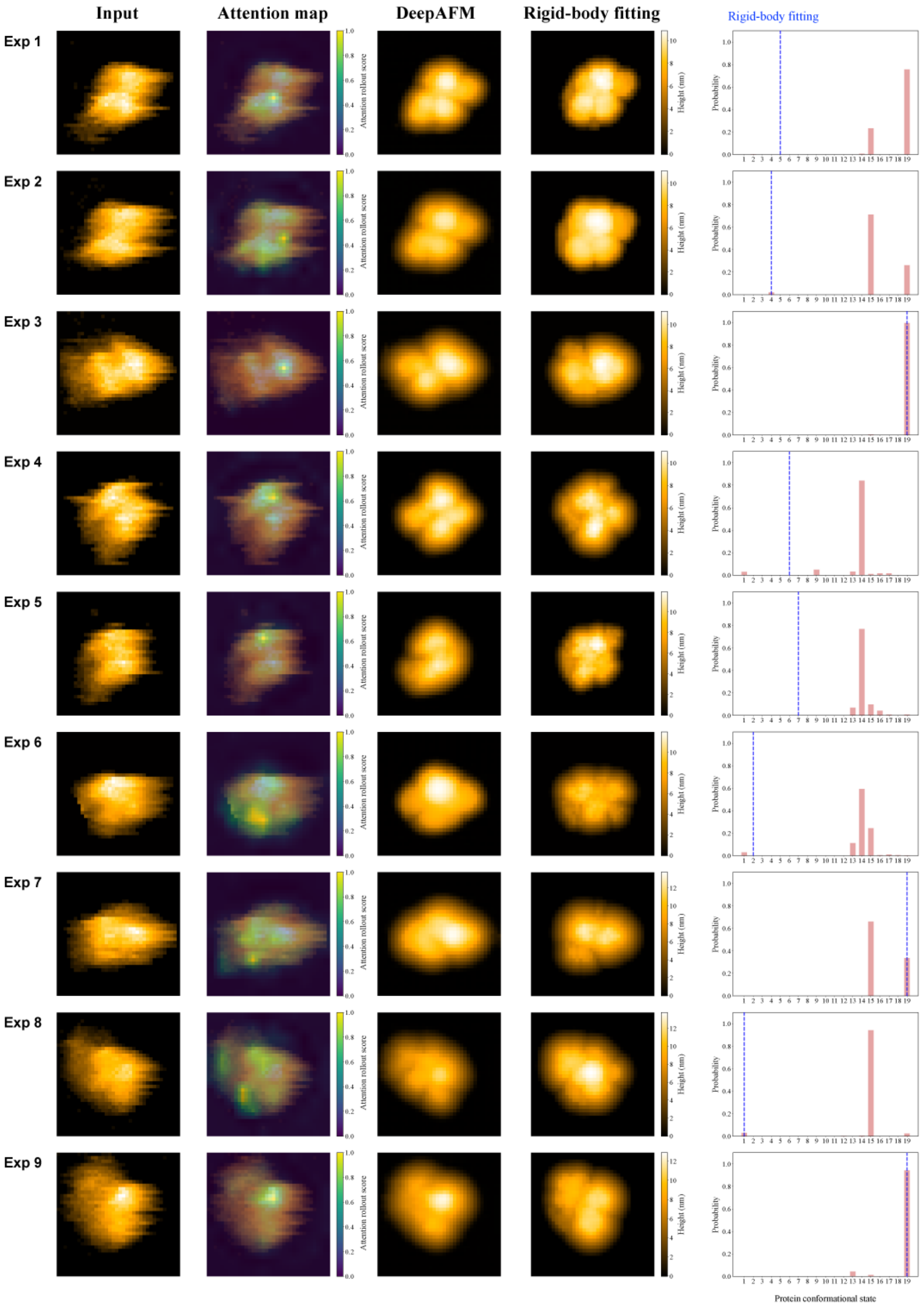

Figure S8. Summary of the experimental data analysis. From left to right: input experimental images, attention maps obtained from DeepAFM, denoised images obtained from DeepAFM, simulated images generated by rigid-body fitting of the 3D structure to the input image, and probability distribution of protein conformational states predicted by DeepAFM. The blue vertical dashed line indicates the conformational state predicted by rigid-body fitting.

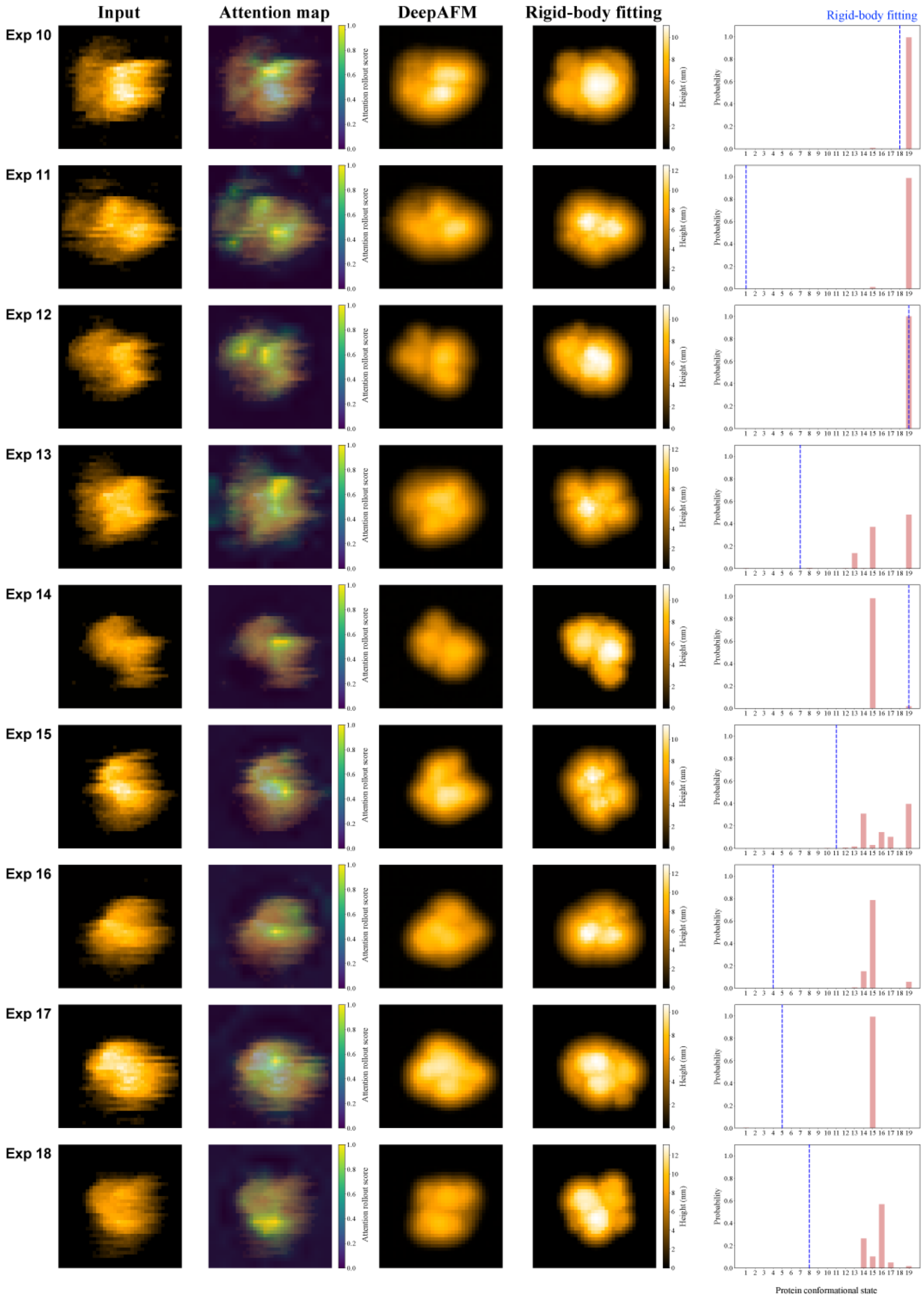

Figure S8. (Continued)

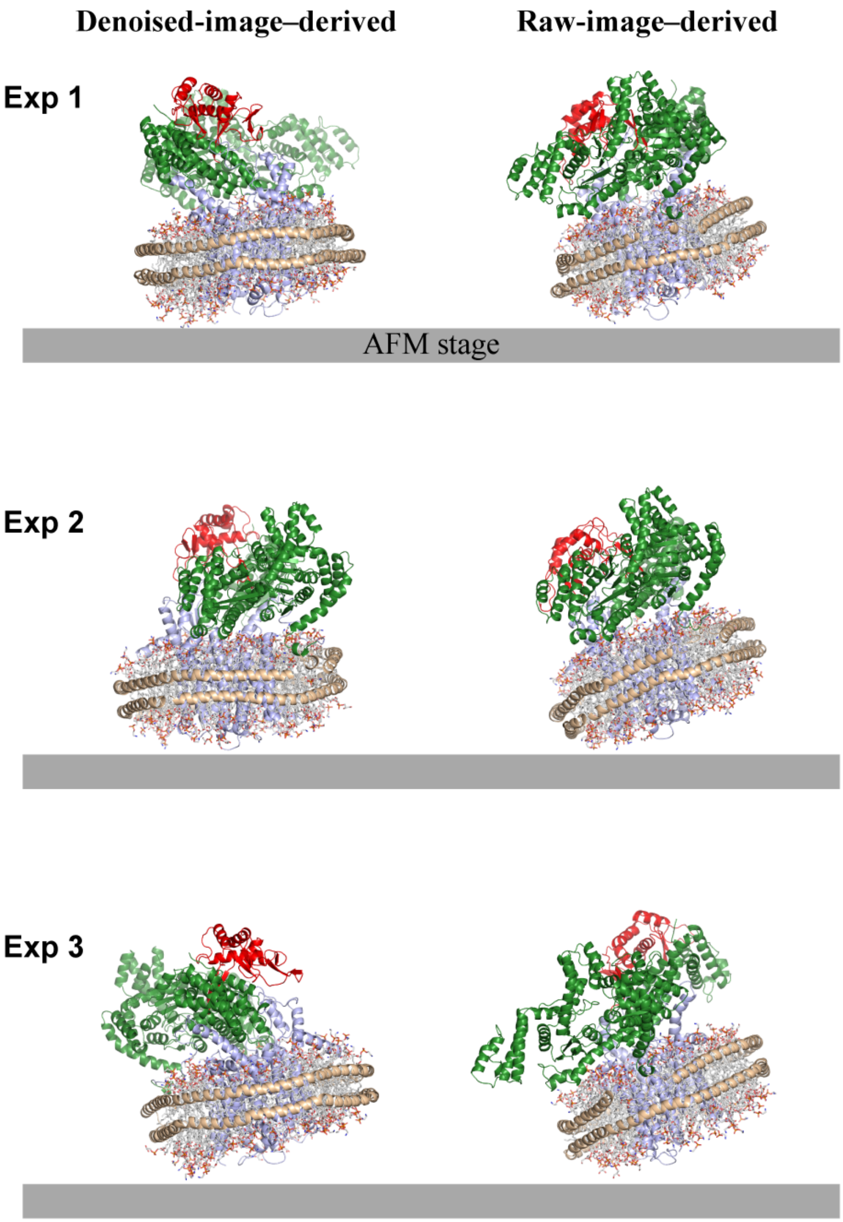

Figure S9. Side views of denoised-image–derived (left) and raw-image–derived (right) 3D structures obtained by rigid-body fitting. From top to bottom, the panels correspond to Exps 1–3 in Figure 6. To clearly show the tilt of the nanodisc relative to the AFM stage, the SecYAEG–ND complex was arbitrarily rotated about the *z*-axis.

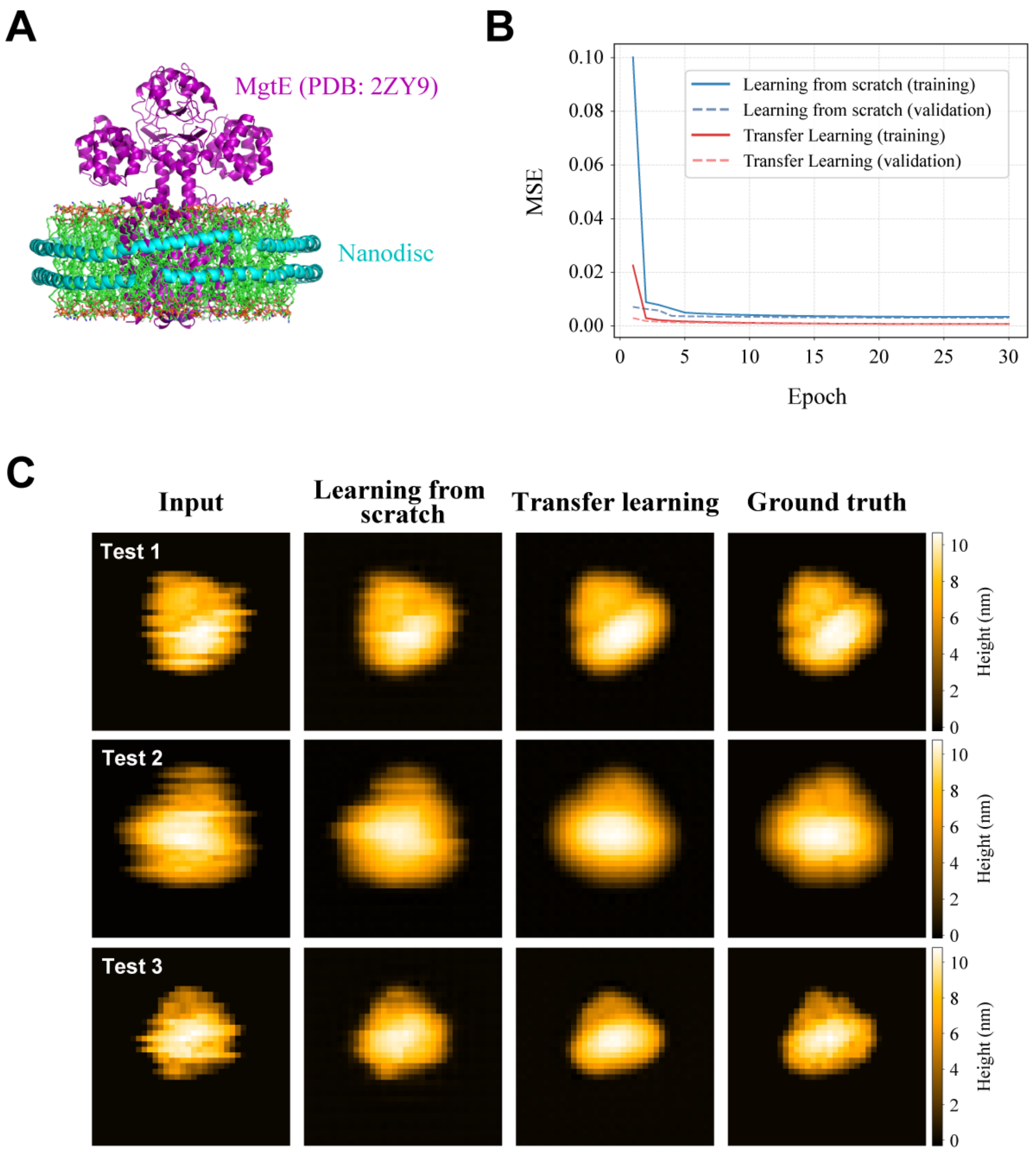

Figure S10. Application of transfer learning to the MgtE–ND complex and comparison with learning from scratch. (A) Reference structure of MgtE–ND. (B) Learning curves of the mean squared error (MSE). (C) Representative denoising examples; from left to right: input images, denoised images obtained by learning from scratch, denoised images obtained by transfer learning, and ground-truth images. MgtE–NDs were placed in a side-on orientation. Simulated images were generated following the SecYAEG–ND case. The AFM tip radius R was randomly selected from 1.0–3.0 nm (0.1 nm increments) and the cone half-apex angle Θ from 5–30° (1° increments). The rotation angles ϕ and θ were randomly sampled from −20° to 20° in 1° increments, whereas ψ from −180° to 180° in 1° increments. A total of 10,000 images generated from the reference structure (panel A) were split into training, validation, and test sets (8:1:1), and the model was trained for 30 epochs. The image resolution was 36 × 36 pixels (0.8 nm/pixel).

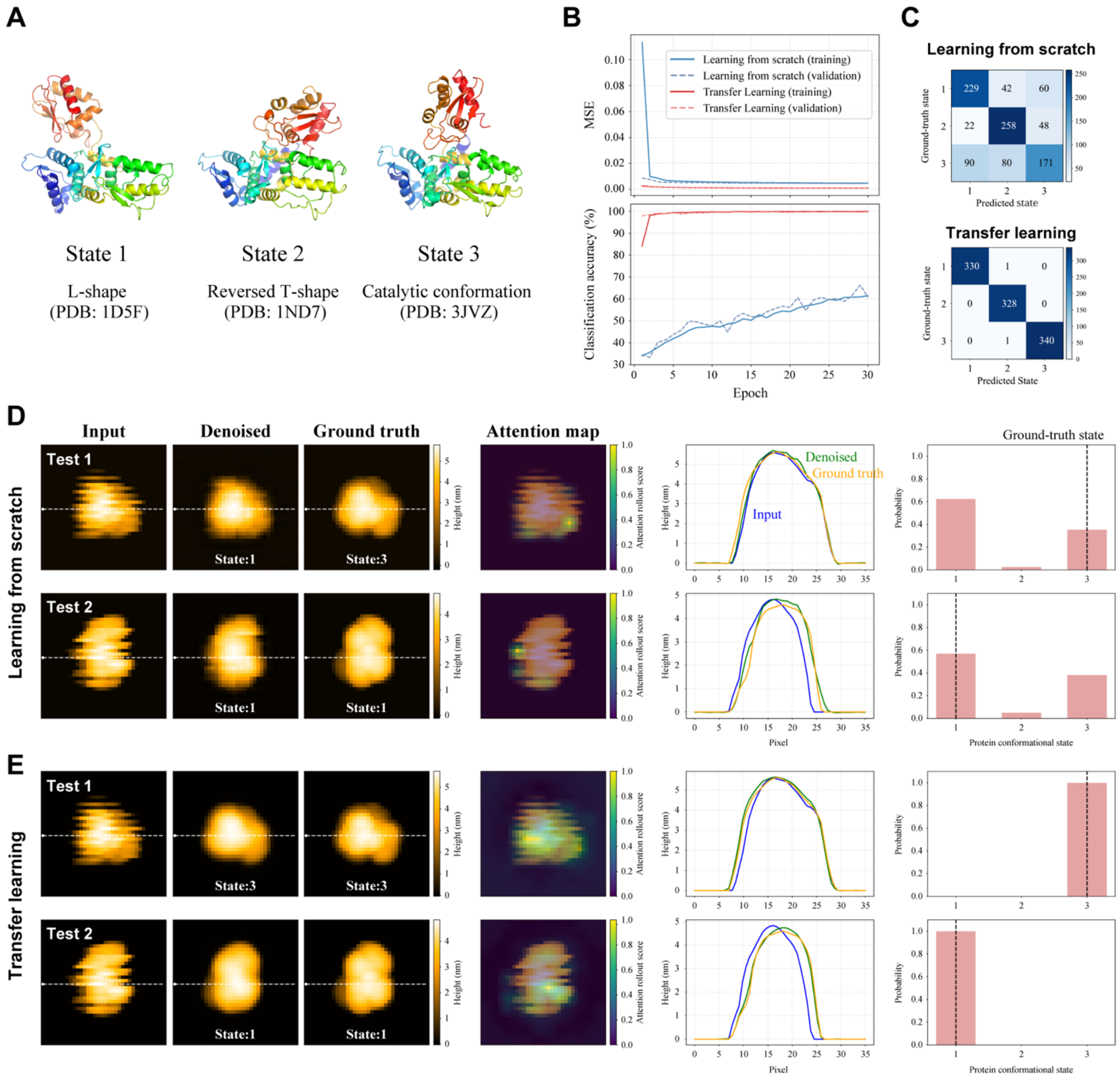

Figure S11. Application of transfer learning to the HECT domain and comparison with learning from scratch. (A) Reference structures of the HECT domain. (B) Learning curves for the MSE and classification accuracy. (C) Confusion matrix. (D, E) Representative results; from left to right: input images, denoised images, ground-truth images, attention maps, height profiles extracted along the white dotted lines in the corresponding images, and class probability distributions obtained from learning from scratch (D) and transfer learning (E). HECT was oriented to ensure that the AFM probe was accessible to both the N-lobe and C-lobe. Simulated images were generated following the SecYAEG–ND case. A total of 10,000 images generated from the three distinct structures (panel A) were split into training, validation, and test sets (8:1:1), and the model was trained for 30 epochs. The image resolution was 36 × 36 pixels (0.6 nm/pixel).

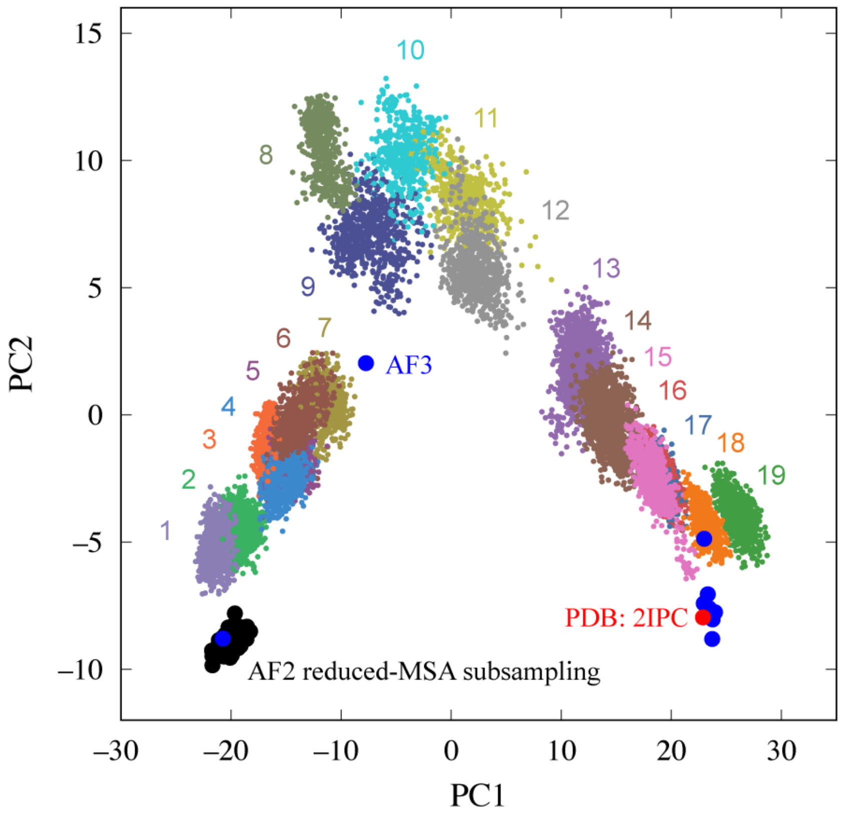

Figure S12. PCA projection of *T. thermophilus* SecY–SecA structures. Structures predicted by AlphaFold 3 (blue circles), AlphaFold 2 reduced-MSA subsampling (black circles), and a docked SecY–SecA model constructed by fitting the X-ray crystal structure of *T. thermophilus* SecA in the wide-open state (PDB ID: 2IPC) to SecY (red circles) are projected onto the PCA map derived from the MD trajectories.

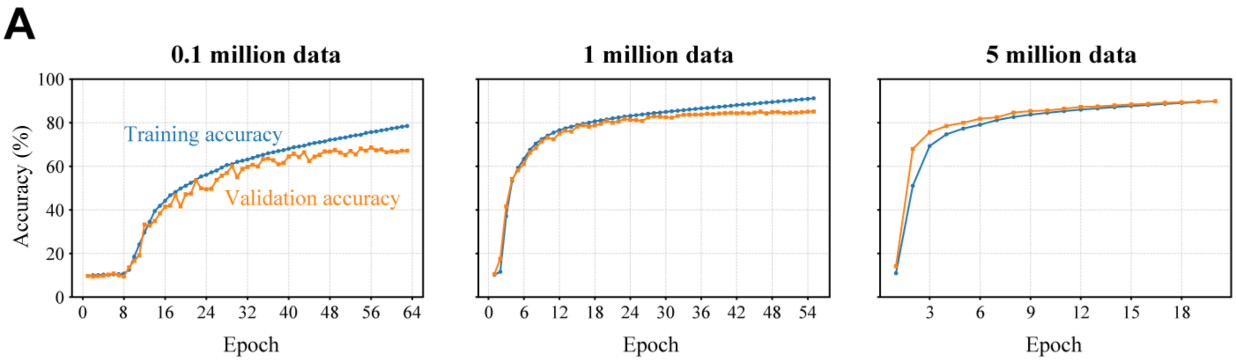

B

	0.1 million	1 million	5 million
**Total loss**	0.9681	0.4574	0.3018
**Classification accuracy (%)**	70.1	87.2	92.1
**Average MSE (nm^2)**	0.225	0.131	0.116
**Average MAE (nm)**	0.232	0.147	0.136

Figure S13. Comparison of model accuracy with training datasets of 0.1 million, 1 million, and 5 million images. (A) Accuracy evolution during training. (B) Final test accuracy. For each dataset size, the training, validation, and test datasets were split at an 8:1:1 ratio.
